# Supplementary material for: A reference panel for linkage disequilibrium and genotype imputation using whole-genome sequencing data from 2,680 participants across India
Source: HGG Adv. 2026 Feb 7;7(2):100579. doi: 10.1016/j.xhgg.2026.100579 (PMC12945573; doi:10.1016/j.xhgg.2026.100579)
Supplement: Document S1. Figures S1–S9 and Table S1 [file mmc1.pdf]

HGGA, Volume 7

Supplemental information

A reference panel for linkage disequilibrium and genotype imputation using whole-genome sequencing data from 2,680 participants across India

Zheng Li, Wei Zhao, Xiang Zhou, Yuk Yee Leung, Gerard D. Schellenberg, Li-San Wang, Sebastian Schönherr, Lukas Forer, Christian Fuchsberger, Sharmistha Dey, Jinkook Lee, Jennifer A. Smith, Aparajit B. Dey, and Sharon L.R. Kardia

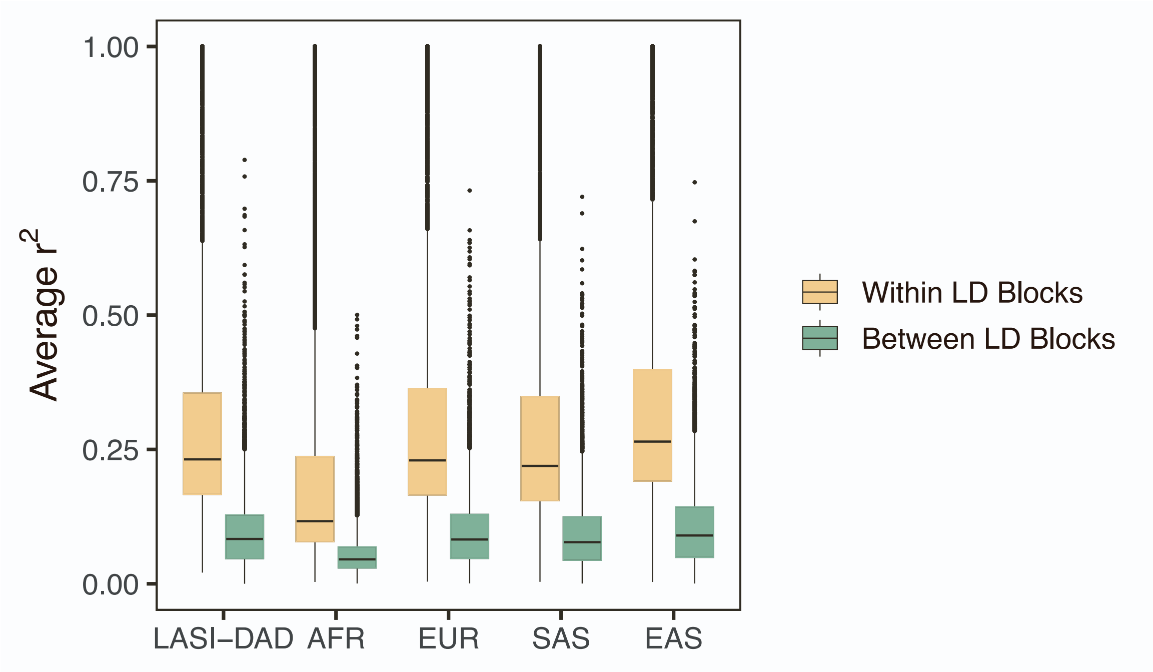

Figure S1. Distribution of average linkage disequilibrium (LD) on chromosome 1. The average LD was evaluated either between all pairs of SNPs within each LD block or between SNPs in each LD block and its adjacent two blocks. LD blocks were identified by BigLD at fine scales. LD was evaluated as the squared correlation coefficient (r^2) of genotypes between a SNP pair.

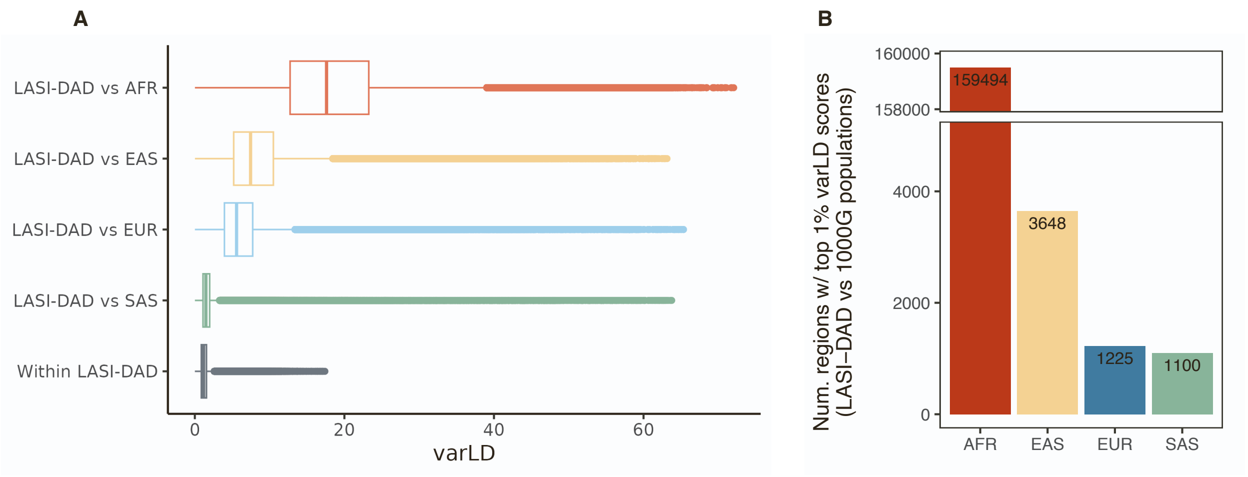

Figure S2. **Extensive differences in linkage disequilibrium (LD) patterns between LASI-DAD and other populations demonstrated by the variation in LD (varLD) scores**. (**A**) Boxplots showing the distribution of varLD scores evaluated between LASI-DAD and each of the four super-populations from 1000G, as well as between two random splits of LASI-DAD samples. The two splits of samples were obtained by randomly selecting 500 samples as the first split and assigning the remaining samples to the second split. (**B**) Number of genomic regions within the top one percentile of the varLD scores across all comparisons between LASI-DAD and each of the four 1000G super-populations.

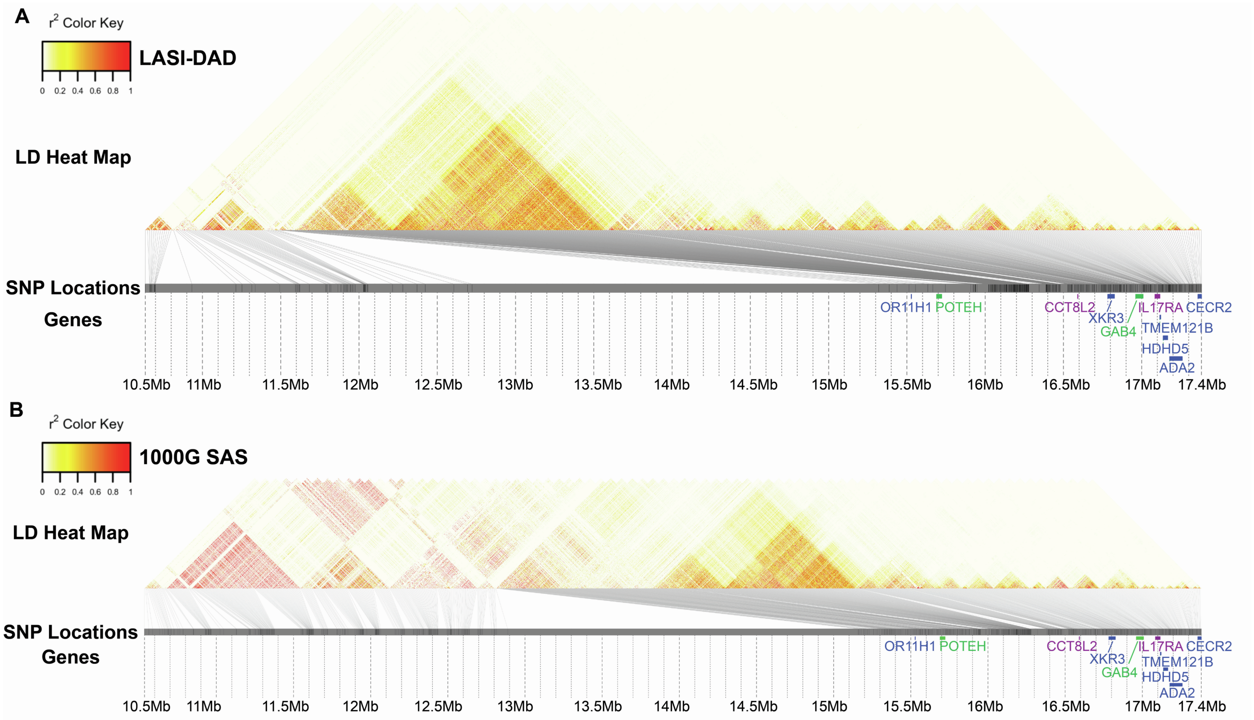

Figure S3. Linkage disequilibrium (LD) patterns of an example genomic region. Visualization of an example LD block identified by LDetect from LASI-DAD in the (**A**) LASI-DAD or (**B**) 1000G SAS population. The LD block spans the region from 10,526,445 to 17,381,714 on chromosome 22 and was selected because it contains the genomic region with the largest variation in LD (varLD) score between LASI-DAD and the 1000G SAS population on this chromosome (varLD = 21.7).

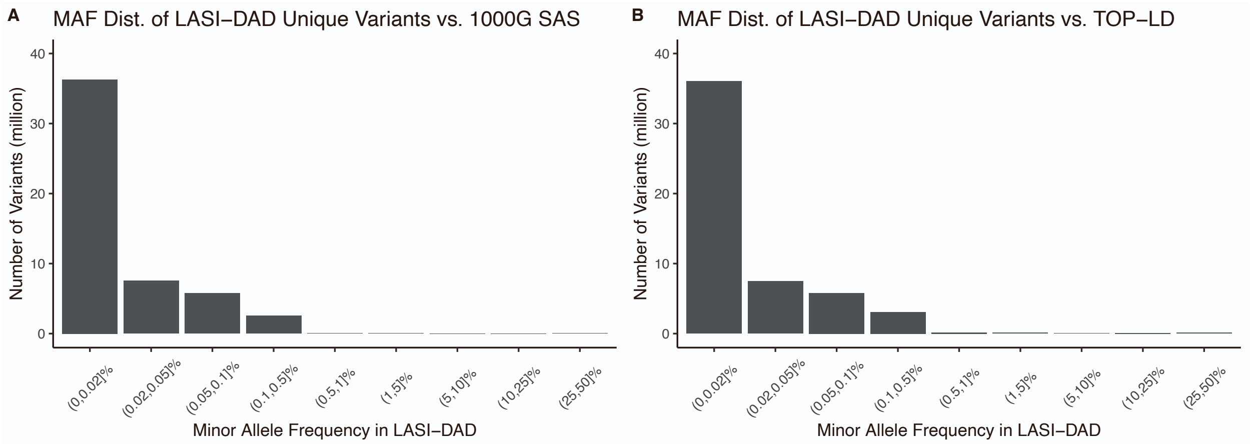

Figure S4. Barplots showing the number of unique variants in the LASI-DAD linkage disequilibrium (LD) lookup panel compared with the (**A**) 1000G SAS panel and (**B**) TOP-LD SAS panel across different minor allele frequency (MAF) ranges.

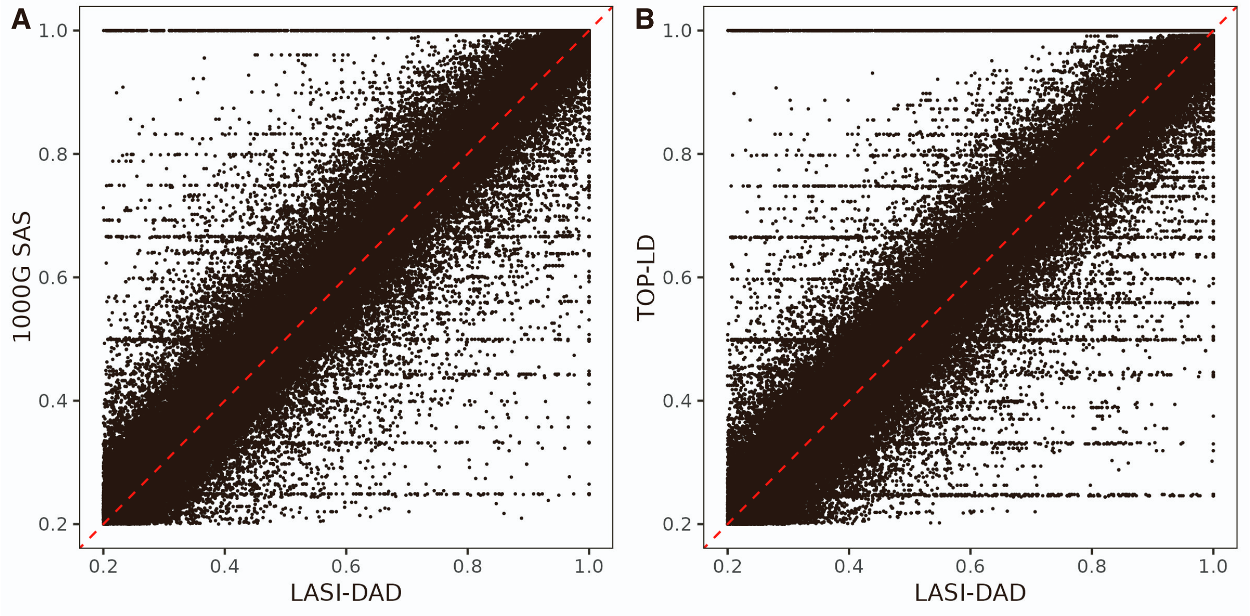

Figure S5. Comparing the linkage disequilibrium (LD) lookup panels constructed with different populations. Scatterplots comparing the r^2 estimates between (A) LASI-DAD and 1000G SAS LD panels and (B) LASI-DAD and TOP-LD SAS LD panels. For computational efficiency, 100,000 variant pairs shared between the two panels were randomly selected from chromosome 1 for the comparison.

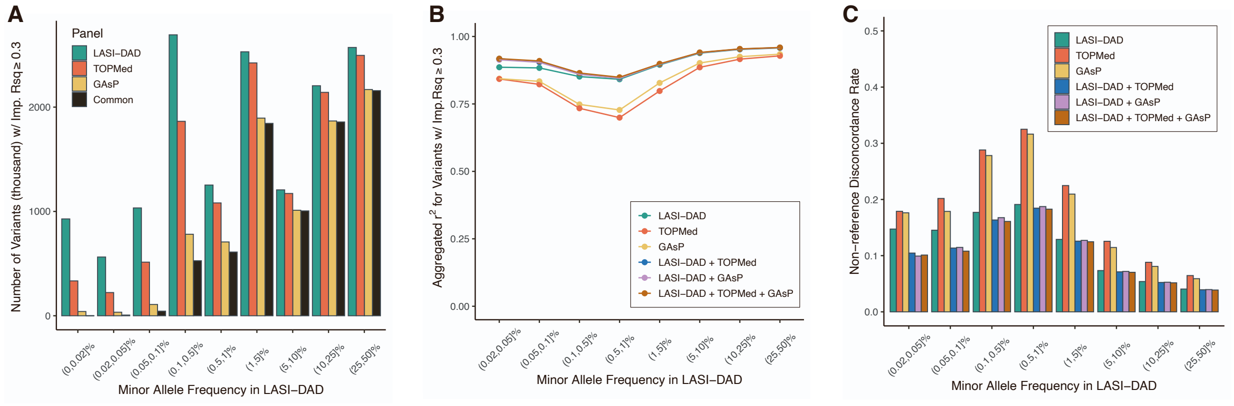

Figure S6. Evaluating LASI-DAD as a reference panel for genotype imputation for variants with an estimated imputation quality score Rsq ≥ 0.3. Compared reference panels include LASI-DAD, TOPMed, and GAsP. (**A**) Bar plots showing the number of genetic variants in LASI-DAD that can be imputed by each reference panel and by all three panels. (**B**) Imputation accuracy was evaluated for each reference panel, including meta-imputation to integrate the imputation results from either LASI-DAD and TOPMed, LASI-DAD and GAsP, or all three panels, across different minor allele frequency (MAF) ranges. The MAF was calculated within the full 2,680 LASI-DAD samples. To ensure a fair comparison, the imputation accuracy was evaluated using the common set of variants that can be imputed by all three panels. Aggregated r^2 was used as the evaluation metric, calculated by grouping variants within a specified MAF range, stacking their imputed dosages and genotype calls from the sequencing data into two separate vectors, and then computing the squared correlation coefficient between the two vectors. (**C**) Imputation accuracy was evaluated based on the nonreference discordance (NRD) rate.

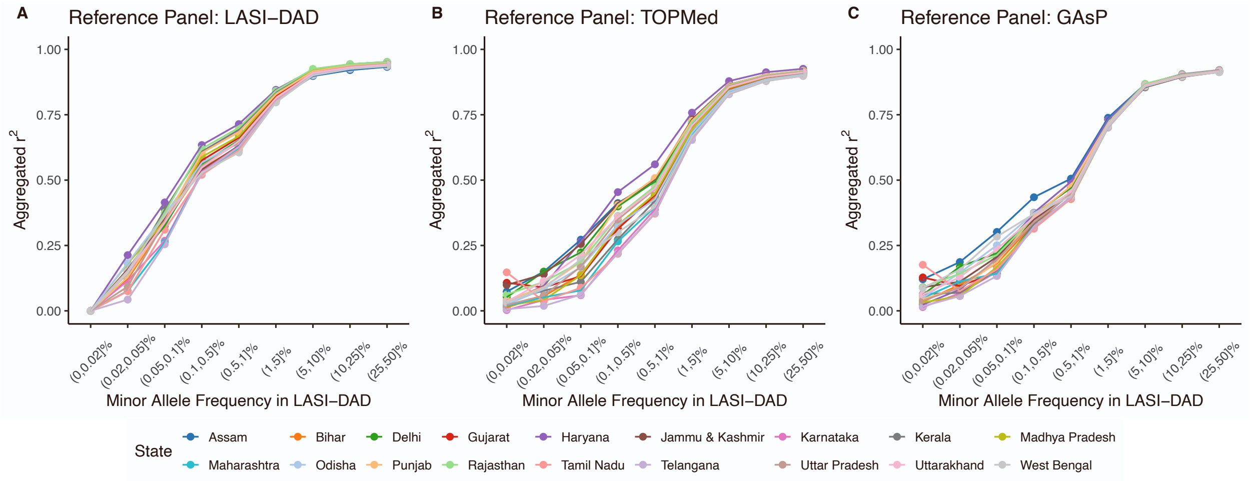

Figure S7. Imputation accuracy by Indian state/territory. Imputation accuracy was evaluated for samples from each Indian state or union territory across different minor allele frequency (MAF) ranges. Compared imputation reference panels include LASI-DAD, TOPMed, and GAsP. The MAF was calculated within the full 2,680 LASI-DAD samples. To ensure a fair comparison, the imputation accuracy was evaluated using the common set of variants that can be imputed by all three panels. Aggregated r^2 was used as the evaluation metric, calculated by grouping variants within a specified MAF range, stacking their imputed dosages and genotype calls from the sequencing data into two separate vectors, and then computing the squared correlation coefficient between the two vectors.

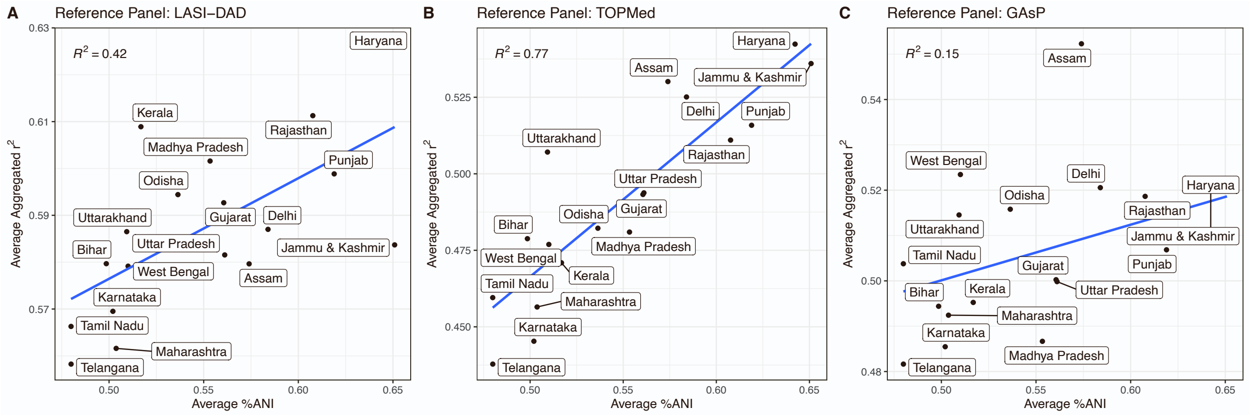

Figure S8. Average proportion of Ancestral North Indian (%ANI) in each state/territory of India versus average aggregated imputation r^2 with different panels. The aggregated r^2 was computed with imputed dosages from panel (**A**) LASI-DAD, (**B**) TOPMed, or (**C**) GAsP.

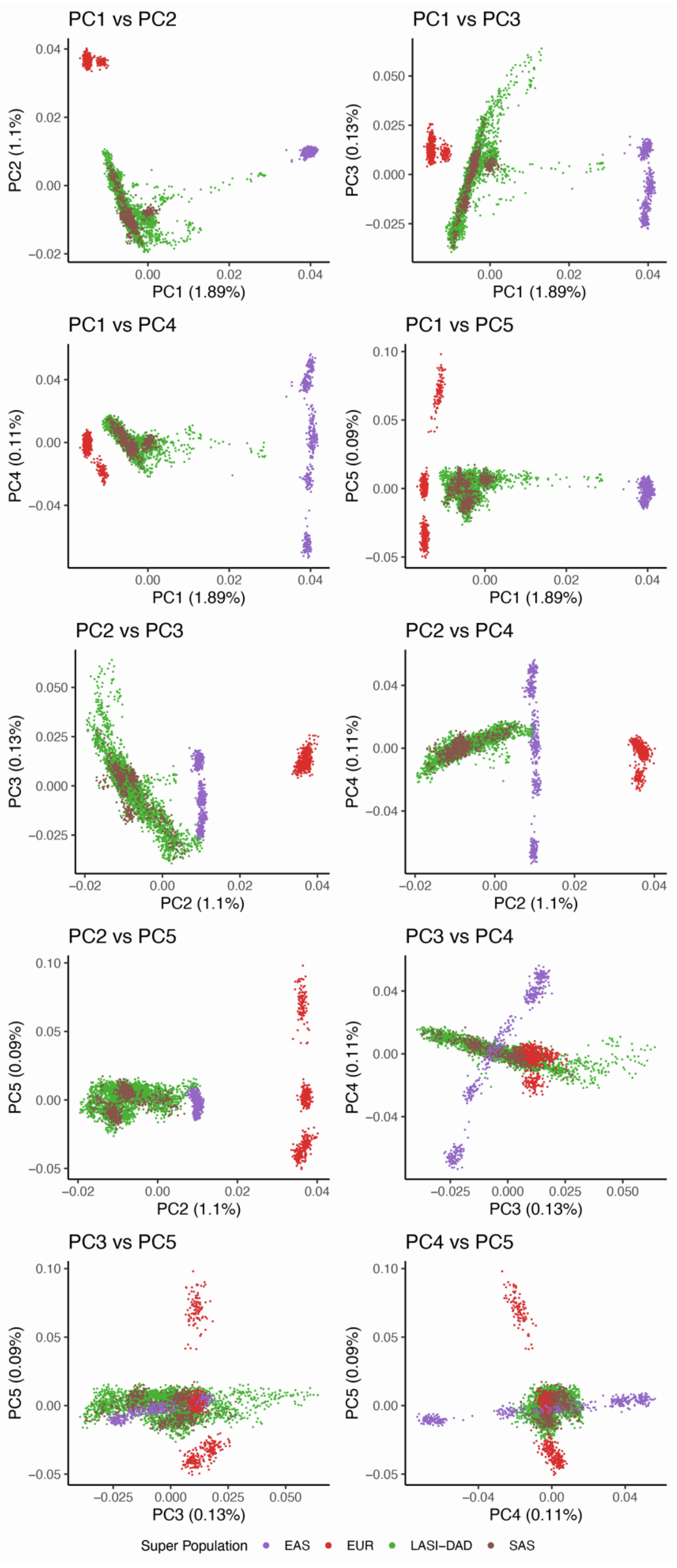
PC1 vs PC2
PC1 vs PC3
PC1 vs PC4
PC1 vs PC5
PC2 vs PC3
PC2 vs PC4
PC2 vs PC5
PC3 vs PC4
PC3 vs PC5
PC4 vs PC5
PC1 (1.89%)
PC2 (1.1%)
PC3 (0.13%)
PC4 (0.11%)
PC5 (0.09%)
Super Population
EAS
EUR
LASI-DAD
SAS

Figure S9. Principal component analysis (PCA) plots. PCA was conducted using Indian samples from LASI-DAD along with European, South Asian, and East Asian samples from 1000G. The proportion of variance explained by each PC is indicated in the parentheses.

Geographic region of India	State/Territory	Sample size
North	Jammu & Kashmir	90
	Punjab	112
	Haryana	171
	Delhi	120
	Rajasthan	151
Northeast	Assam	72
East	Bihar	137
	West Bengal	187
	Odisha	216
Central	Uttarakhand	68
	Uttar Pradesh	154
	Madhya Pradesh	77
West	Gujarat	228
	Maharashtra	185
South	Telangana	171
	Karnataka	122
	Tamil Nadu	159
	Kerala	260

Table S1. Sample sizes of Indian states and union territories by geographic region in LASI-DAD.
