# Supplementary material for: A reference panel for linkage disequilibrium and genotype imputation using whole-genome sequencing data from 2,680 participants across India
Source: HGG Adv. 2026 Feb 7;7(2):100579. doi: 10.1016/j.xhgg.2026.100579 (PMC12945573; doi:10.1016/j.xhgg.2026.100579)
Supplement: Document S2. Article plus supplemental information [file mmc3.pdf]

HGG Advances

ARTICLE

A reference panel for linkage disequilibrium and genotype imputation using whole-genome sequencing data from 2,680 participants across India

Zheng Li,[1,10] Wei Zhao,[2,3,10,12,*] Xiang Zhou,[1] Yuk Yee Leung,[4] Gerard D. Schellenberg,[4] Li-San Wang,[4] Sebastian Schönherr,[5] Lukas Forer,[5] Christian Fuchsberger,[6] Sharmistha Dey,[7] Jinkook Lee,[8] Jennifer A. Smith,[2,3,11,*] Aparajit B. Dey,[9,11] and Sharon L.R. Kardia[3,11]

Summary

India is the most populous country globally, yet genetic studies involving Indian individuals remain limited. The Indian population is composed of many founder groups and has a mixed genetic ancestry, including an ancestral component not observed anywhere outside of India. This presents a unique opportunity to uncover novel disease variants and develop tailored medical interventions. To facilitate genetic research in India, a crucial first step is to create a foundational resource that serves as a benchmark for future population studies and methods development. Thus, we constructed the largest and most nationally representative linkage disequilibrium (LD) and genotype imputation reference panels in India to date, using high-coverage whole-genome sequencing data of 2,680 participants from the Longitudinal Aging Study in India-Harmonized Diagnostic Assessment of Dementia (LASI-DAD). As an LD reference panel, LASI-DAD includes 69.5 million variants, representing 170% and 213% increases relative to the 1000 Genomes Project and TOP-LD South Asian panels, respectively. Besides serving as an LD lookup panel, LASI-DAD facilitates various statistical analyses relying on precise LD estimates. In polygenic risk score (PRS) analyses, LASI-DAD improved the PRS predictive performance by 2.1%–35.1% across traits and studies. As an imputation reference panel, LASI-DAD enhanced imputation accuracy, measured by the Pearson correlation between imputed and true genotypes, by 3%–101% (mean 38%) compared with the TOPMed panel and by 3%–73% (mean 27%) compared with the Genome Asia Pilot panel across different allele frequencies. The LASI-DAD reference panel is publicly available to benefit future studies.

Introduction

India, with a population of over 1.4 billion, is home to more than 4,500 anthropologically defined groups that are characterized by a rich diversity in languages, religions, and cultures.[1] The Indian population largely descends from a mixture of two genetically divergent populations: Ancestral North Indians (ANI), who are related to Central Asians, Middle Easterners, and Europeans, and Ancestral South Indians (ASI), who are not related to any groups outside the subcontinent and are only distantly related to the indigenous Andaman Islanders.[2,3] Due to this unique ASI component, the Indian population has a distinctive genetic structure and harbors many unique variants not present elsewhere. Thus, genetic variants identified from populations in other areas of the world may not have the same effects in Indians, and many variants specific to the Indian population could play significant roles in predisposing this population to certain diseases. Furthermore, due to consanguineous and endogamous marriages, the ancestral Indian population experienced founder events that are more extreme than Ashkenazi Jews and Finnish populations, which led to many genetically diverse subgroups.[4] As a result, the frequency of deleterious or protective variants may increase substantially, leading to increased power for gene mapping. All of these highlight the critical need and potential for new discovery of variants (otherwise rare outside India) from genetic studies in this population. Despite the need and opportunity, India has been largely underrepresented in genetic studies, making up <2% of human study participants in the catalog of genome-wide association studies (GWASs catalog).[5,6] This lack of representation hinders the discovery of novel disease variants and the development of more tailored medical interventions or treatments in the Indian population.

To facilitate genetic research in India, a crucial first step is to create an Indian-representative reference panel that can serve as a resource for future studies in India as well as methods development. This resource may serve as a

[1]Department of Biostatistics, School of Public Health, University of Michigan, Ann Arbor, MI, USA; [2]Survey Research Center, Institute for Social Research, University of Michigan, Ann Arbor, MI, USA; [3]Department of Epidemiology, School of Public Health, University of Michigan, Ann Arbor, MI, USA; [4]Penn Neurodegeneration Genomics Center, Department of Pathology and Laboratory Medicine, Perelman School of Medicine, University of Pennsylvania, Philadelphia, PA, USA; [5]Institute of Genetic Epidemiology, Medical University Innsbruck, Innsbruck, Austria; [6]Eurac Research, Institute for Biomedicine, Via Volta 21, Bolzano, Italy; [7]Department of Biophysics, All India Institute of Medical Sciences, New Delhi, India; [8]Department of Economics, University of Southern California, Los Angeles, CA, USA; [9]Venu Geriatric Institute, New Delhi, India
[10]These authors contributed equally
[11]Senior author
[12]Lead contact
*Correspondence: zhaowei@umich.edu (W.Z.), smjenn@umich.edu (J.A.S.)
https://doi.org/10.1016/j.xhgg.2026.100579.
© 2026 The Author(s). Published by Elsevier Inc. on behalf of American Society of Human Genetics.
This is an open access article under the CC BY license (http://creativecommons.org/licenses/by/4.0/).

reference panel for linkage disequilibrium (LD) and genotype imputation for the Indian population. LD refers to the non-random co-occurrence of alleles at different genomic loci.[7] A robust and representative estimate of genome-wide LD statistics for a given population is critical for a wide range of genetic analyses,[8–12] such as statistical fine-mapping and polygenic risk score (PRS) construction. Genotype imputation, however, estimates unobserved genotypes in samples with sparse microarray data and has been widely used in GWASs to boost power, fine-map candidate causal variants, and facilitate meta-analysis across studies.[13] Extensive efforts have been made by several initiatives such as the 1000 Genomes Project (1000G), Trans-Omics for Precision Medicine (TOPMed), and the Genome Asia Pilot (GAsP) project to establish reference panels.[14–17] However, these reference panels have included a limited number of Indian samples and/or primarily focused on expatriate communities outside of India. For example, 1000G includes 489 unrelated individuals from South Asia, with only 103 Gujarati Indians in Houston, Texas and 102 Indian Telugu individuals in the United Kingdom. Likewise, the only South Asian representation in TOPMed comes from the Pakistani Risk of Myocardial Infarction Study, with a sample size of 644. While GAsP consists of a broad range of populations across Asia with a total sample size of 1,739, only 598 individuals are from India. This limited sample size and representation has restricted the utility of these resources to serve as reference panels for the Indian population.

In the present study, we have constructed the largest and most nationally representative LD and genotype imputation reference panel in India to date, using high-coverage (30×) whole-genome sequencing (WGS) data from 2,680 Indian participants from the Longitudinal Aging Study in India-Harmonized Diagnostic Assessment of Dementia (LASI-DAD). For the LD reference panel, we characterized LD patterns in LASI-DAD by identifying LD blocks and evaluating regional differences in LD between LASI-DAD and four super-populations from 1000G. Given the genetic heterogeneity in this population, we further characterized and compared LD patterns by sub-populations within LASI-DAD and evaluated how such differences manifest in PRS performance across sub-populations. We further assessed the utility of this LD reference panel in facilitating PRS construction in South Asians. The LASI-DAD LD reference panel covers a substantially higher number of genetic variants in India than previous panels and facilitates various statistical analyses that rely on precise LD estimates. To evaluate LASI-DAD as an imputation reference panel, we performed imputation using phased LASI-DAD WGS data or other widely used reference panels, including TOPMed and GAsP, and compared the imputation accuracy across the spectrum of minor allele frequencies. As an imputation reference panel, LASI-DAD enables the imputation of many Indian-specific variants that are not present in the other panels with improved imputation accuracy.

Material and methods

LASI-DAD

LASI is a nationally representative survey of more than 70,000 adults aged 45 years and older in India that aims to understand the health, economic, and social well-being of older adults in India. LASI-DAD is an ancillary study of LASI that subsampled adults who were at least 60 years of age from 18 states and union territories across India to specifically focus on late-life cognition and dementia. Participants were selected based on a two-stage stratified sampling approach that oversampled individuals with a high risk of cognitive impairment.[18] Briefly, participants in the larger LASI cohort were first classified into those at high risk or low risk of cognitive impairment based on cognitive testing or on the proxy report for those who did not complete the cognitive tests. Finally, an approximately equal number of participants were randomly sampled from the high-risk and low-risk strata, with a target sample size for each state/territory proportional to the population size.

A total of 2,762 LASI-DAD participants who consented to blood sample collection were carried forward for WGS at MedGenome (Bangalore, India) at an average read depth (DP) of 30×. Genotype calling and quality control (QC) of the raw WGS data were conducted at the Genome Center for Alzheimer's Disease at the University of Pennsylvania. Specifically, samples were excluded due to low sequencing coverage, sample contamination, sex mismatch, discordance with previous genotyping array data,[19] unexpected duplications, or serving as technical controls. A total of 2,680 samples were retained in the analysis after sample-level QC. The number of samples by state/territory is reported in Table S1, with states/territories grouped by geographic regions. For genotype-level QC, each genotype was examined and set to missing if its DP was <10 or genotype quality score was <20. We focused on bi-allelic variants and excluded multi-allelic variants from the analysis. There was a total of 84,270,926 bi-allelic variants prior to QC. For variant-level QC, a variant was excluded if it fell in the 99.8% Variant Quality Score Recalibration (VQSR) tranche or higher (7,901,516 variants), had a call rate ≤80% (1,274,464 additional variants), was monomorphic (1,497,930 additional variants), or had an average depth >500 reads (0 additional variants). We further excluded 2,487,055 variants in low-complexity regions as identified by mdust.[20] After variant-level QC, a total of 71,109,961 bi-allelic variants that included 66,204,161 autosomal single-nucleotide polymorphisms (SNPs) and 4,905,800 indels were retained in the analysis. We phased the genotype data with Eagle2.4[21,22] without a reference panel following the user manual of Eagle2.4 (https://alkesgroup.broadinstitute.org/Eagle/#x1-130004). Finally, we estimated genetic principal components (PCs) using PC Analysis in Related Samples (AiR) in both LASI-DAD and the combined samples of LASI-DAD and 1000G, as described previously.[23,24]

Characterizing and comparing LD patterns between LASI-DAD and 1000G super-populations

We characterized LD patterns by identifying LD blocks and evaluating the variation in LD (varLD) scores. For both LD block identification and varLD score calculation, we focused on a total of 2,607 unrelated samples based on a kinship coefficient threshold of $2^{-3.5}$ (or 0.088) obtained from PC-Relate.[25] LD blocks represent segments of the genome where genetic variants are often inherited together. The identification of LD blocks can be useful

in many ways for genetic studies. For example, some PRS methods estimate SNP effects one block at a time to achieve a good balance between modeling accuracy and computational efficiency.[26–28] As another example, local genetic correlation analysis quantifies the genetic similarity of complex traits within individual LD blocks.[29–31]

We applied LDetect[32] to identify approximately independent LD blocks in LASI-DAD and compare with those identified in four super-populations from 1000G: African (AFR), East Asian (EAS), European (EUR), and South Asian (SAS). LDetect first partitions each chromosome into smaller chunks to facilitate parallel processing. For each chunk, it computes a covariance matrix between all pairs of SNPs using the shrinkage estimator from Wen and Stephens.[33] Notably, both steps require the input of a recombination map to refine the boundary of chunks and to adjust the level of shrinkage. Following MacDonald et al.,[34] we used the recombination map inferred for Gujarati Indians in Houston, Texas ($n = 113$) in the 1000G phase 3 data.[35] Recombination rates were linearly interpolated to all SNPs in the LASI-DAD data. Afterward, LDetect converts the covariance matrix into a vector, with each element representing the sum of elements along a corresponding antidiagonal of the matrix. Boundaries of LD blocks appear as "dips" in the vector and can be identified by first smoothing the vector and then searching for local minima. In the analysis, we focused on SNPs with minor allele frequencies (MAFs) $\geq$1% and set the average block size to 7,000 SNPs following MacDonald et al.[34] To compare LASI-DAD and the 1000G super-populations, we directly obtained LD blocks from MacDonald et al.[34] Notably, the American super-population was not included by MacDonald et al.,[34] likely due to its extensive genetic admixture.

While LDetect is effective in capturing large LD blocks, it is not well suited for identifying finer LD structures within each block, which can provide additional insights into local LD variation. To further identify LD blocks at a finer scale, we applied Big-LD.[36] Big-LD proceeds by first identifying LD clusters through a modified clique-based clustering (CLQ) algorithm. Each cluster consists of SNPs that are in strong LD with each other, yet are not necessarily physically consecutive. Big-LD then expands each LD cluster to form a consecutive genomic segment that includes all SNPs inside the region. Finally, by merging mutually overlapping segments based on interval graph modeling, Big-LD effectively partitions the genome into a set of finer LD blocks. Big-LD was implemented in the gpart R package (version 1.17.0). We set the |r| threshold to be its default value of 0.5 in the CLQ algorithm, where |r| represents the absolute correlation coefficient between the dosage values of a SNP pair. For the comparison of LD blocks at the fine scale, we applied Big-LD to the same four super-populations in 1000G. Specifically, we obtained the genotype data for 3,202 samples from the 1000G 30× dataset on GRCh38 (https://www.internationalgenome.org/data-portal/data-collection/30x-grch38) and restricted the samples to 2,504 unrelated individuals as reported by 1000G for our analysis.[15] Following MacDonald et al.,[34] we excluded the African Caribbean in Barbados ($n = 96$) and African Ancestry in Southwest US ($n = 61$) from the AFR population due to genetic admixture, as well as the Finnish in Finland ($n = 99$) from the EUR population, due to genetic isolation. The total number of samples included was 504 for AFR, 504 for EAS, 404 for EUR, and 489 for SAS.

In addition to LD block identification, we compared LD patterns by evaluating the varLD score between LASI-DAD and the four 1000G super-populations.[37] varLD examines regional patterns of LD between two populations by comparing the signed r^2 values across all pairs of SNPs in a genomic region with an MAF $\geq$1% that are not strand ambiguous in both populations, where r^2 represents the squared correlation coefficient between the dosage values of a SNP pair. Specifically, for a particular genomic region, we first calculated the LD matrix for the two populations, defined as a SNP-by-SNP correlation matrix, with each matrix element representing the signed r^2 value between a SNP pair. We then obtained the eigenvalues of the LD matrix from each population and calculated the varLD score as the sum of the absolute differences between the corresponding eigenvalues derived from the two populations. We employed a sliding-window approach and calculated varLD scores within consecutive windows of 50 SNPs. Each consecutive window was obtained by shifting the current window by one SNP in the direction of the forward strand.[25]

Characterizing LD patterns by LASI-DAD sub-population

In addition to comparisons with 1000G super-populations, we evaluated LD patterns across three LASI-DAD sub-populations, defined according to the population structure in India. The population structure in India is correlated with geography, with the majority of the individuals lying along a north/south cline with varying proportions of ANI and ASI ancestries. ANI is genetically related to West Eurasians while ASI is related (distantly) to indigenous Andaman Islanders.[2] In contrast, individuals in the east of India are considered out-of-cline due to admixture with additional ancestral populations such as EAS. Following Kerdoncuff et al.,[38] we clustered individuals into those that lie along the north/south cline and those that are out of the cline. For individuals that lie along the north/south cline, we estimated the proportion of ANI ancestry (%ANI) using the f_4-statistic implemented in the ADMIXTOOLS R package (version 1.3.0).[39] The f_4-statistic measures the excess of West Eurasian ancestry in a test individual relative to the Onge population based on an Indian population model described by Moorjani et al.[3] Finally, we grouped individuals from LASI-DAD into three sub-populations based on their respective cline and %ANI values to facilitate Indian sub-population analysis. The three sub-populations included 945 individuals in the high %ANI group ($0.540 \leq$ %ANI <0.824), 957 in the low %ANI group (%ANI ≤ 0.540), and 778 that were out of the north/south cline. We conducted this analysis on the set of unrelated samples, including 935, 941, and 731 individuals in the respective groups. For simplicity, we focused on the evaluation of varLD scores in the sub-population analysis.

Transferability of PRSs

A PRS captures an individual's genetic predisposition to a complex trait or disease and is often calculated as the sum of SNP genotypes weighted by their effect sizes estimated from GWASs. However, most GWASs have been conducted in individuals of EUR ancestry,[40] and PRSs derived from these GWASs may be substantially less predictive in other genetic ancestries.[11] We evaluated the transferability of the PRS derived from GWASs of primarily EUR ancestry to LASI-DAD individuals. Specifically, we obtained PRS weights for height and body mass index (BMI) from the Genetic Investigation of Anthropometric Traits (GIANT) consortium. For height, we estimated PRS weights by applying PRS-continuous shrinkage (CS) to a cross-ancestry GWAS meta-analysis[41] (https://portals.broadinstitute.org/collaboration/giant/index.php/GIANT_consortium_data_files). The meta-analysis included 5,380,080 individuals across 281

studies from the GIANT consortium and 23andMe, with 75.8% of the individuals being primarily of EUR ancestry and 1.4% being primarily of SAS ancestry. For BMI, we estimated PRS weights by applying PRS-CS[26] to a GWAS meta-analysis of GIANT consortium studies and UK Biobank.[42] The meta-analysis included ~700,000 individuals of primarily EUR ancestry. After obtaining the PRS weights, we evaluated the performance of PRS in the three LASI-DAD sub-populations (high %ANI, low %ANI, and out-of-cline) using incremental R^2. Following Yengo et al.,[41] we calculated the incremental R^2 for height by first centering and standardizing height to have a mean of zero and a variance of one within each sex. The incremental R^2 was then calculated as the difference in the coefficient of determination between two linear regression models: one that includes age and the top 20 genotype PCs as covariates and the other that additionally includes the height PRS, with height as the response variable in both models. For BMI, we followed a similar procedure to calculate the incremental R^2. Additionally, we estimated the 95% confidence interval (CI) for the incremental R^2 with bootstrapping. For each LASI-DAD sub-population under evaluation, we resampled individuals with replacement and recalculated the incremental R^2. This procedure was repeated 1,000 times, and the 2.5th and 97.5th percentiles of the resulting bootstrap estimates were taken as the lower and upper bounds of the 95% CI.

Providing LASI-DAD as a reference panel for LD

A robust and representative estimate of genome-wide LD statistics for a given population plays an essential role in a wide range of genetic analyses.[8–12] As the largest and most nationally representative WGS study of the Indian population, LASI-DAD has the potential to provide more accurate estimates of LD with higher coverage of genetic variants, serving as a valuable resource for LD in this population. We evaluated the utility of LASI-DAD WGS data as an LD reference panel to facilitate both LD lookup and various statistical analyses that rely on precise LD estimates. We constructed an LD reference panel using 2,607 unrelated individuals from the LASI-DAD WGS data and provided it as a public resource to facilitate future research in the community. Specifically, for all pairs of genetic variants within 1 Mb of one another, we computed the squared Pearson correlation coefficient (r^2) between the phased haplotypes, D′, and the direction of the Pearson correlation coefficient. We reported LD statistics for pairs of variants that have an r^2 statistic ≥0.2. Of note, we did not use a minimum minor allele count to exclude any variants. We compared these LD statistics with those obtained from the SAS populations in 1000G and TOP-LD.[16] TOP-LD estimated LD statistics for 239 selected SAS individuals from TOPMed WGS data.[14] We obtained the LD statistics for the TOP-LD SAS panel directly from its website (http://topld.genetics.unc.edu/about.php). For the 1000G SAS panel, we computed the LD statistics following procedures similar to those used for LASI-DAD and TOP-LD. Of note, an LD panel for the GAsP is not publicly available, therefore it was not included in this comparison.

In addition to serving as an LD lookup panel, LASI-DAD facilitates various statistical analyses that rely on precise LD estimates. To simplify data sharing while ensuring privacy protection, many statistical methods can directly make use of GWAS summary statistics as input.[43–46] These summary statistics typically include marginal Z scores for each SNP and a SNP-SNP correlation matrix. The SNP-SNP correlation matrix is commonly referred to as the LD matrix and can be estimated from a reference panel consisting of individuals with the same genetic ancestry as the GWAS. In principle, the closer the LD estimates from the reference panel match those in the GWAS population, the better the results are likely to be in the statistical analysis. This often requires the reference panel to be sufficiently large and representative of the GWAS population. To demonstrate the utility of LASI-DAD as an LD reference panel, we conducted a PRS analysis where we compared the predictive performance of PRS constructed with either the LASI-DAD or 1000G SAS LD reference panel. We estimated PRS weights using PRS-CS, which takes GWAS summary statistics and an external LD reference panel as inputs. We obtained summary statistics for height and BMI from four studies of SAS ancestry, including a GWAS of height[41] and an exome-wide association study (ExWAS) of BMI[47] from the GIANT consortium, and GWASs of both height and BMI from the Genes & Health study.[48] The GIANT data included 77,890 individuals of primarily SAS ancestry for height and 29,398 for BMI. The Genes & Health data included 36,317 British Pakistani and British Bangladeshi individuals for height and 34,408 for BMI. The summary statistics included marginal association results for 1,339,059 and 246,158 SNPs for height and BMI from the GIANT consortium, and 24,241,756 and 23,922,099 bi-allelic variants for height and BMI from Genes & Health. We constructed the LASI-DAD LD reference panel following a procedure similar to that of Ge et al.[26] Specifically, we focused on the 2,607 unrelated samples and filtered out SNPs that have an MAF <1% or are strand ambiguous. We restricted the SNPs to those in the HapMap 3 panel to achieve a good balance between computational efficiency and prediction accuracy. For each LD block as identified by LDetect, we computed a SNP-SNP correlation matrix, with each element in the matrix representing the Pearson correlation coefficient between the dosage values of a SNP pair. For each study and LD reference panel, we applied PRS-CS to estimate PRS weights. We evaluated the performance of PRS in the full 2,680 LASI-DAD samples using incremental R^2, calculated following the same procedure as described above.

Providing LASI-DAD as a reference panel for genotype imputation

Given the unique population structure of India, LASI-DAD has the potential to serve as a valuable resource for genotype imputation to enhance the analysis of array-genotyped samples, especially those collected from the Indian populations. We evaluated the utility of LASI-DAD WGS data as an imputation reference panel and compared it with the GAsP and TOPMed imputation panels. Specifically, we randomly selected 180 samples, 10 from each Indian state or union territory, from the LASI-DAD dataset and selected 530,845 genetic markers that were present on the Illumina Infinium Global Screening Array-24 version 2.0 BeadChip. These 180 samples served as a test dataset to evaluate the imputation accuracy. Following Yu et al.,[49] we evaluated the imputation accuracy by calculating the aggregated squared correlation coefficient (r^2), computed using aggRSquare (https://github.com/yukt/aggRSquare), which groups variants within a specified MAF range, stacks their imputed dosages and genotype calls from the sequencing data into two separate vectors, and then calculates the aggregated r^2 as the squared correlation coefficient between the two vectors. As a sensitivity analysis, we evaluated the imputation accuracy for variants with an estimated imputation quality score Rsq ≥0.3. As another sensitivity analysis, we also evaluated the imputation performance by calculating the nonreference discordance (NRD) rate,[50] expressed as $NRD = (e_{rr}+e_{ra}+e_{aa})/(e_{rr}+e_{ra}+e_{aa}+m_{ra}+m_{aa})$ with e_{rr}, e_{ra}, and e_{aa}

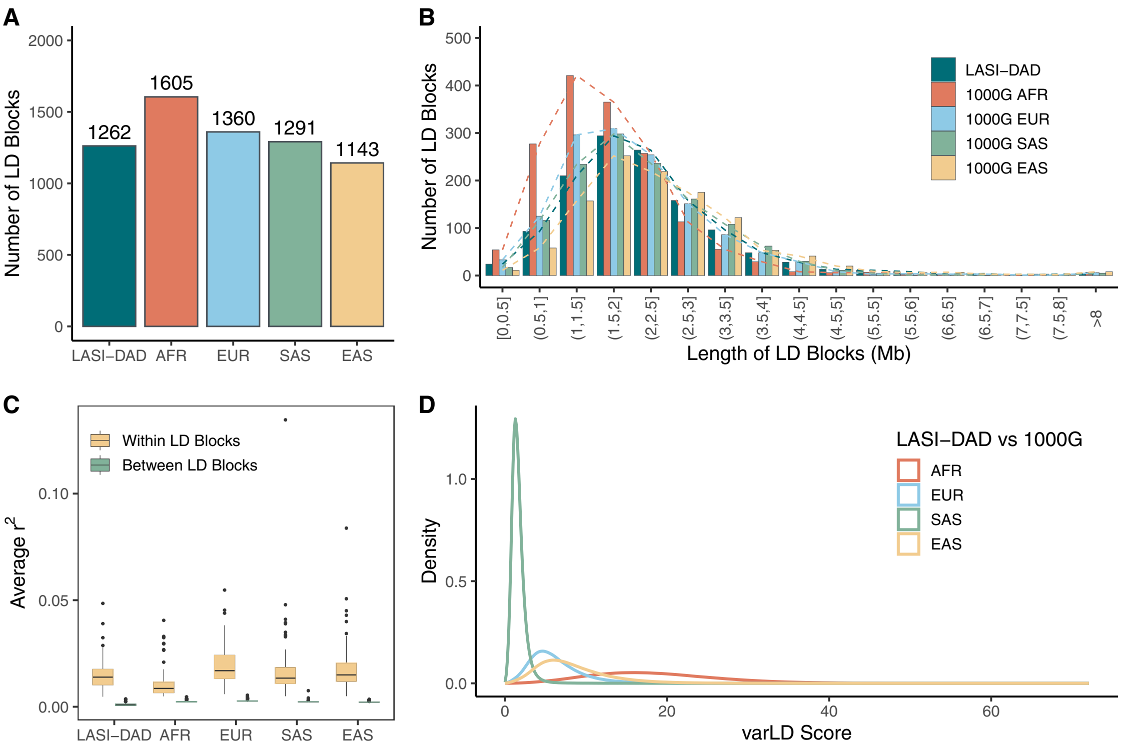

Figure 1. Linkage disequilibrium (LD) patterns in LASI-DAD compared with four super-populations from the 1000 Genomes Project (1000G)
The four super-populations include African (AFR), European (EUR), South Asian (SAS), and East Asian (EAS).
(A) Number of LD blocks identified by LDetect.
(B) Distribution of LD block lengths in millions of base pairs (Mb), with LD blocks grouped into different length ranges for clear visualization.
(C) Boxplots showing the distribution of average LD on chromosome 1, calculated between either all pairs of SNPs within each LD block or SNPs in each LD block and its adjacent two blocks. LD is evaluated as the squared correlation coefficient (r^2) of genotypes between an SNP pair.
(D) Distribution of variation in LD (varLD) scores evaluated between LASI-DAD and each of the four super-populations from 1000G. varLD scores were calculated as the sum of the absolute differences between the corresponding eigenvalues derived from the LD matrices of two comparing populations, using sliding windows of 50 SNPs each.

representing the counts of the mismatches for the homozygous reference and heterozygous and homozygous alternative genotypes, respectively, and m_{ra} and m_{aa} as the counts of the matches for the heterozygous and homozygous alternative genotypes. We imputed unobserved genotypes with Minimac4 (version 1.0.2) using either LASI-DAD ($n = 2{,}500$ after excluding the 180 samples from the test dataset), GAsP ($n = 1{,}654$), or TOPMed ($n = 97{,}256$) as the reference panel. For GAsP and TOPMed, we carried out the imputation directly using the Michigan Imputation Server and the TOPMed Imputation Server,[51] respectively. For LASI-DAD, we followed the same analysis pipeline described on the TOPMed Imputation Server for data QC and imputation. Briefly, we split each chromosome into chunks of 10 Mb, filtered out chunks with <3 variants, and excluded monomorphic variants. We then conducted genotype imputation using Minimac4 with the same parameter settings as those used by the imputation server. In addition to the imputation with a single reference panel, we performed meta-imputation by combining imputation results from LASI-DAD with those from GAsP, TOPMed, or both using MetaMinimac2.[49] We evaluated the imputation accuracy across different ranges of MAF, where the MAFs were calculated among all 2,680 LASI-DAD samples. To facilitate future research in the community, the LASI-DAD reference panel is now available on the Michigan Imputation Server.

Ethics statement

Investigations were conducted in accordance with the principles outlined in the Declaration of Helsinki. The study was approved by the institutional review boards at the University of Southern California (UP-15-00684) and the University of Michigan (HUM00166956). Informed consent was obtained from all participants.

Results

Characterizing LD patterns in the Indian population from LASI-DAD

We characterized LD patterns in LASI-DAD by identifying LD blocks and evaluating the varLD scores.[37] For LD block identification, we first applied LDetect,[32] which has been widely used by various statistical methods. In total, we identified 1,262 blocks in LASI-DAD, which is comparable to the 1,291 blocks identified in the SAS population from 1000G (Figure 1A). For the other 1000G populations, the number of identified blocks was 1,605, 1,360, and 1,143 for AFR, EUR, and EAS, respectively (Figure 1A). As expected, we observed the largest number of LD blocks in

the AFR population as Africans are the oldest population ancestrally, and there has been more time for recombination to break down the LD structure.[52] On average, the length of LD blocks was 2.2 Mb in LASI-DAD and 1.7, 2.1, 2.2, and 2.5 Mb in the AFR, EUR, SAS, and EAS populations from 1000G, respectively (Figure 1B). Notably, the average r^2 for variant pairs within blocks was 0.015, while the average r^2 for variant pairs across neighboring blocks was 0.001 on chromosome 1 in LASI-DAD (Figure 1C). These results indicate that LDetect captures LD structure at a broad scale, characterized by wide genomic regions that are largely independent of one another and exhibit relatively weak linkage within each region. The comparable number and size of such LD blocks between LASI-DAD and 1000G SAS suggest a similar LD structure at the broad scale.

To better understand the LD structure at a finer scale, we further applied Big-LD.[36] As expected, the number of LD blocks identified by Big-LD was much larger and ranged from 96,415 to 143,383 across the 5 populations (Table S2). For these LD blocks, we observed a considerably higher average r^2 both within and between blocks, with average values of 0.32 and 0.098, respectively, in LASI-DAD (Figure S1). Although LASI-DAD more closely resembles the 1000G SAS population than the other global populations, it was notably different from the 1000G SAS. Specifically, LASI-DAD presents a smaller number of blocks (106,383 in LASI-DAD vs. 108,751 in 1000G SAS; Table S2) and a larger median block size (5.9 kb in LASI-DAD vs. 5.4 kb in 1000G SAS; Table S2), suggesting stronger LD at the finer scale in LASI-DAD relative to the 1000G SAS.

In addition to LD block identification, we evaluated regional differences in LD between pairs of populations with varLD scores.[37] As anticipated, the smallest LD difference was observed between LASI-DAD and the 1000G SAS population, with an average varLD score of 1.67 (Figure 1D). This was followed by the EUR (6.21), EAS (8.34), and AFR (18.5) populations. Despite the lowest average varLD score between LASI-DAD and 1000G SAS, a considerable number of genomic regions exhibit high varLD scores, as evidenced by the large number of data points above the interquartile range (Figure S2A). This indicates that in specific regions of the genome, notable LD differences are present between LASI-DAD and the 1000G SAS population. Furthermore, we quantified the number of genomic regions with notable LD differences by identifying regions in the top 1% of varLD scores across all comparisons between LASI-DAD and each of the four 1000G populations (Figure S2B). Interestingly, the number of such genomic regions is similar between the comparison of LASI-DAD vs. SAS (1,100) and LASI-DAD vs. EUR (1,225). Finally, we visualized an LD block from LDetect with the largest varLD score on chromosome 22 in the comparison between LASI-DAD and 1000G SAS (Figure S3). Although this region corresponds to a single LD block in both panels, the underlying local LD structure varies substantially between LASI-DAD and 1000G SAS. These results suggest that the LD structure of the Indian population is not identical to that represented by the 1000G SAS panel and that LASI-DAD more accurately captures the LD structure unique to the Indian population.

Indian population structure and transferability of PRS

In addition to comparisons with 1000G super-populations, we evaluated LD patterns across the three LASI-DAD sub-populations (Figures 2A and 2B; Table S1). We found that the overall differences in LD across LASI-DAD sub-populations were modest compared with those observed between LASI-DAD and the 1000G EUR, EAS, and AFR populations but comparable to those between LASI-DAD and the 1000G SAS population (Figure 2C vs. Figure 1D). The average varLD scores were 2.10, 1.81, and 1.69 between the out-of-cline and high %ANI groups, high %ANI and low %ANI groups, and the out-of-cline and low %ANI groups, respectively (Figure 2C). Of note, the average varLD scores were of a rank similar to that of the relative genetic distances calculated as Euclidean distance between the median centroids of clusters in the PC space (Figures 2B and 2C). The relative genetic distances were 0.008, 0.008, and 0.003 between the out-of-cline and high %ANI groups, high %ANI and low %ANI groups, and the out-of-cline and low% ANI groups, respectively. In general, a larger genetic distance corresponds to a greater difference in LD.

Despite overall modest differences in LD across LASI-DAD sub-populations, such differences can still impact the performance of various statistical analyses, such as the PRS. We evaluated the transferability of a height and BMI PRS derived from GWASs of primarily EUR ancestry to LASI-DAD individuals. We found that the predictive performance of PRS varied across LASI-DAD sub-populations (Figure 2D), with relative performance consistent with their genetic relatedness to the EUR population (Figures 2A and 2B). As expected, PRS performed the best in the high %ANI group due to its closer relatedness to the EUR population, achieving a prediction accuracy of 17% for height (95% CI: 11%–20%) and 6% for BMI (95% CI: 3.4%–8.8%), as measured by incremental R^2. In contrast, the prediction accuracy was 14% (95% CI: 10%–18%) and 3% (95% CI: 1.1%–4.7%) in the low %ANI group and 10% (95% CI: 5.3%–14%) and 2% (95% CI: 0.41%–4.1%) in the out-of-cline group for height and BMI, respectively. Notably, the height PRS performed significantly better in the high %ANI group compared with the out-of-cline group, while BMI PRS performed significantly better in the high %ANI group compared with both the low %ANI and out-of-cline groups. Despite the variation across sub-populations, the overall prediction accuracy in LASI-DAD remained substantially lower than that in the EUR population, where the incremental R^2 reached as high as 44.7% for height[41] and 10.2% for BMI.[42] The limited transferability of PRS in LASI-DAD

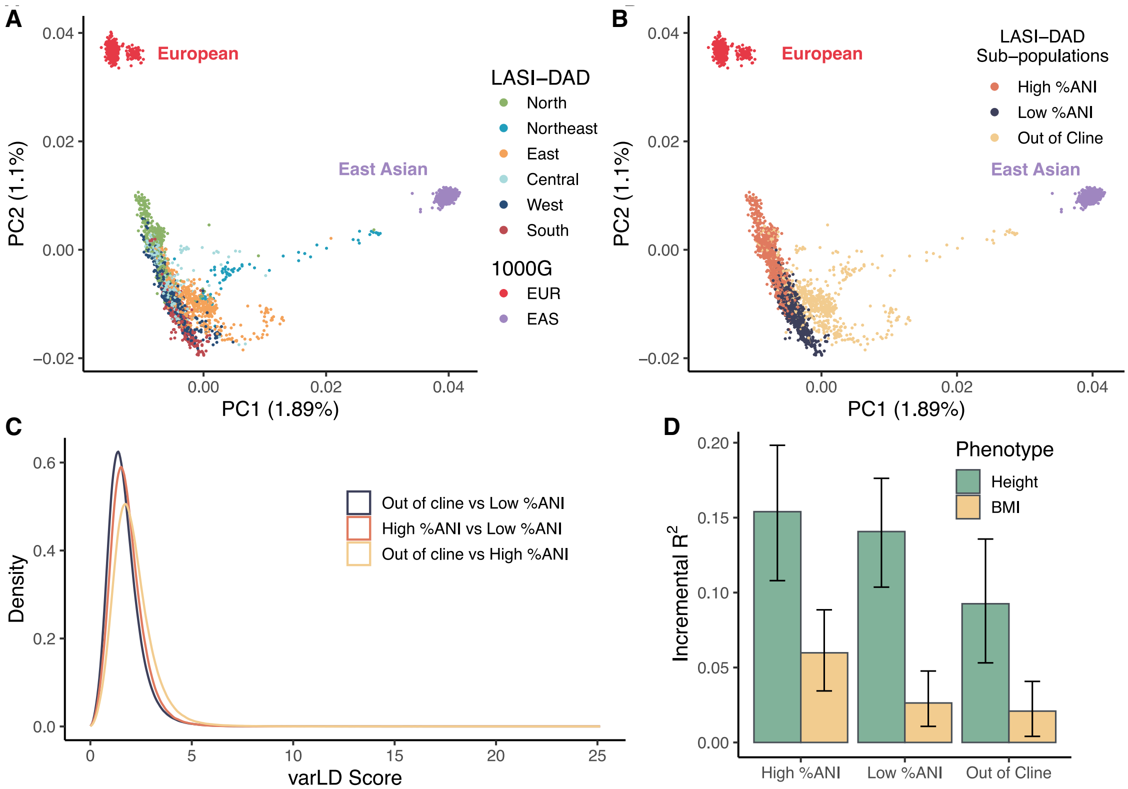

Figure 2. Linkage disequilibrium (LD) patterns in Indian sub-populations from LASI-DAD

Individuals from LASI-DAD were grouped into three sub-populations based on their proportion of Ancestral North Indian ancestry (%ANI) and presence on the north/south cline of India to facilitate Indian sub-population analysis. The three sub-populations included the high %ANI group ($0.540 \leq$ %ANI < 0.824; $n = 935$), low %ANI group (%ANI ≤ 0.540; $n = 941$), and the out-of-the north/south cline group ($n = 731$).

(A) Principal-component analysis (PCA) plot illustrating the genetic relationship among Indian, European (EUR), and East Asian (EAS) populations. PCA was conducted using Indian samples from LASI-DAD, along with EUR, SAS, and EAS samples from 1000G. The proportion of variance explained by each PC is indicated in parentheses. LASI-DAD individuals are colored by their geographic region of birth in India to highlight the relationship between population structure and geography in India.

(B) The same PCA plot with LASI-DAD individuals colored by their sub-population groups.

(C) Distribution of variation in LD (varLD) scores evaluated between LASI-DAD sub-populations. varLD scores were calculated as the sum of the absolute differences between the corresponding eigenvalues derived from the LD matrices of two comparing populations, using sliding windows of 50 SNPs each.

(D) Predictive performance, measured by incremental R^2, of polygenic risk scores (PRSs) derived from GWAS of primarily EUR ancestry in Indian sub-populations in LASI-DAD. Evaluated traits include height and body mass index (BMI), with GWAS summary statistics obtained from the GIANT consortium. Error bars indicate 95% confidence intervals estimated from bootstrapping with 1,000 resamples.

hinders its clinical utility, especially for South and East Indian populations that are more distantly related to the EUR population and benefit less from its large sample size. These findings suggest that more efforts need to be made toward genetic research in this unique population to reduce potential health disparities.[40]

Providing LASI-DAD WGS data as an LD reference panel

We evaluated the utility of LASI-DAD WGS data as an LD reference panel to facilitate both LD lookup and various statistical analyses that rely on precise LD estimates. In the LD lookup panel, LASI-DAD included a total of 69.5 million variants, representing a 170% (43.8 million) and 213% (47.3 million) increase in variant coverage relative to the 1000G SAS and TOP-LD SAS panels, respectively (Figure 3A). To be included in the panels, these variants have at least one tagged variant with $r^2 \geq 0.2$. As expected, the major increase in variant coverage is observed in the low MAF spectrum (e.g., MAF <1%) (Figure 3A). The majority of variants unique to LASI-DAD are extremely rare (Figure S4) and fall below the detection thresholds of the other two panels (~0.1% in the 1000G SAS panel and ~0.2% in the TOP-LD SAS panel, based on their respective sample sizes). This suggests that the increased number of variants in LASI-DAD is primarily due to its substantially larger sample size and the resulting greater power to detect rare variants. Interestingly, we found that the number of variants with an MAF ≥1% is smaller in the LASI-DAD panel (8.7 million) than in the 1000G SAS (11.6 million) and TOP-LD SAS (9.4 million) panels (Figure 3A). Further analyses

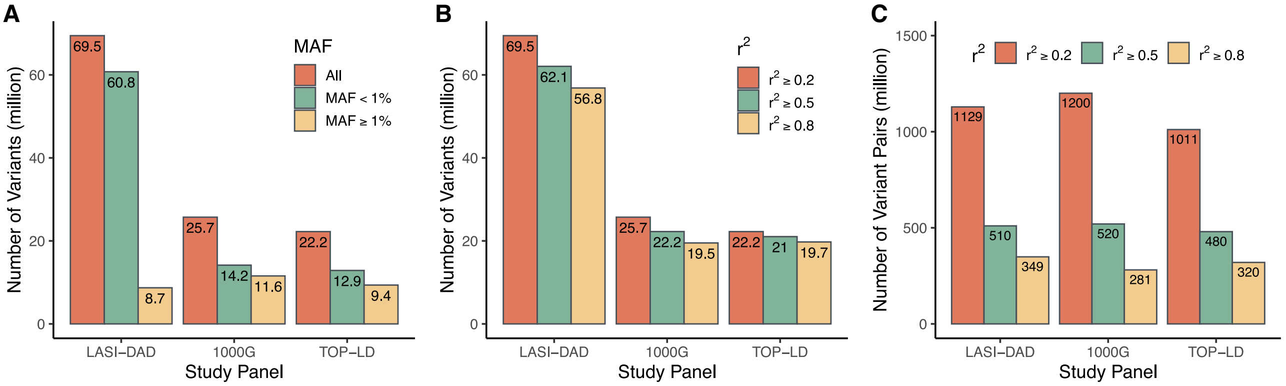

Figure 3. Number of autosomal variants included in different linkage disequilibrium (LD) reference panels
Three LD reference panels were compared, including Indian samples from LASI-DAD ($n = 2{,}607$), South Asian (SAS) samples from 1000G (1000G SAS, $n = 489$), and SAS samples from TOPMed (TOP-LD SAS, $n = 239$).
(A) Number of variants stratified by the range of minor allele frequency (MAF).
(B) Number of variants with at least one other variant in LD under different r^2 cutoffs, where r^2 represents the squared Pearson correlation coefficient between the phased haplotypes of a variant pair.
(C) Number of variant pairs stratified by different r^2 cutoffs.

revealed that 377,720 and 478,767 common variants (MAF $\geq$1%) in 1000G SAS and TOP-LD SAS, mostly near the MAF = 1% cutoff, have an MAF <1% in LASI-DAD. This likely reflects the enhanced accuracy in estimating low-frequency variants as a result of the substantially larger sample size in LASI-DAD. Meanwhile, 2,819,660 and 824,693 common variants in 1000G SAS and TOP-LD SAS, respectively, are absent from LASI-DAD. Of these, ~22% of the 1000G SAS and 36% of the TOP-LD SAS variants failed the LASI-DAD QC protocol, which was more stringent than the other two panels. For example, LASI-DAD applied a 99.8% VQSR filter that excluded a significant number of variants (7,901,516 variants). To our knowledge, 1000G applied a less stringent VQSR threshold of 99.9%, and TOP-LD did not apply such a filter.[16,53] The remaining 78% of the 1000G and 64% of the TOP-LD variants are absent from LASI-DAD because 1000G and TOP-LD included both bi-allelic and multi-allelic variants, whereas LASI-DAD included only bi-allelic variants in the analysis. Therefore, the lower number of common variants in LASI-DAD is primarily due to protocol differences. Nonetheless, the coverage of genetic variants remained the highest for the LASI-DAD panel regardless of the cutoff in r^2 (Figure 3B). In addition, most variants in the LD lookup panel have at least one tagged variant with $r^2 \geq 0.8$, with proportions of variants being 82%, 76%, and 89% in the LASI-DAD, 1000G SAS, and TOP-LD SAS panels, respectively (Figure 3B). Notably, the number of variant pairs remained largely similar across different LD lookup panels regardless of the cutoff in r^2 (Figure 3C). This is expected, as the additional variants in LASI-DAD are predominantly rare and generally do not form strong LD with many other variants, and therefore, they contribute relatively few additional variant pairs. Finally, we compared LD estimates across the three lookup panels by examining the common set of variant pairs on chromosome 1. The Pearson correlation of r^2 statistics was 0.94 between LASI-DAD and 1000G SAS, 0.92 between LASI-DAD and TOP-LD SAS, and 0.91 between 1000G SAS and TOP-LD SAS, indicating an overall high consistency across the three panels. However, specific estimates differed considerably between LASI-DAD and the other two panels, as evidenced by the wide band around the red diagonal lines on the scatterplots (Figure S5). These findings again suggest that while LD patterns are broadly similar between LASI-DAD and other SAS panels at a genome-wide level, there is substantial fine-scale heterogeneity in LD structure.

To demonstrate the advantage of LASI-DAD as an LD reference in statistical analyses, we conducted a PRS analysis where we compared the predictive performance of PRS constructed with either the LASI-DAD or 1000G SAS LD reference panel. To obtain PRS weights, we applied PRS-CS[26] to four studies of SAS ancestries, including a GWAS of height[41] and an ExWAS of BMI[47] from the GIANT consortium and GWASs of both height and BMI from the Genes & Health study.[48] We evaluated the prediction accuracy of PRS in LASI-DAD individuals and found that the predictive performance of PRS consistently trends higher with the LASI-DAD panel than with the 1000G SAS panel for all four studies (Figure 4). Specifically, the PRS constructed with the LASI-DAD LD panel explains 4.42% and 0.50% of variance in height and BMI, respectively, using summary statistics from the GIANT consortium. In contrast, the PRS constructed with the 1000G SAS panel explained 3.70% and 0.37% of the variance in the corresponding two traits. The improvement in R^2 was 0.72% (95% CI: 0.21%–1.27%) for height and 0.13% (95% CI: −0.13% to 0.43%) for BMI. When using summary statistics from the Genes & Health study, the PRS constructed with the LASI-DAD panel explained 2.33% and 1.44% of variance in height and BMI, respectively. In comparison, the PRS

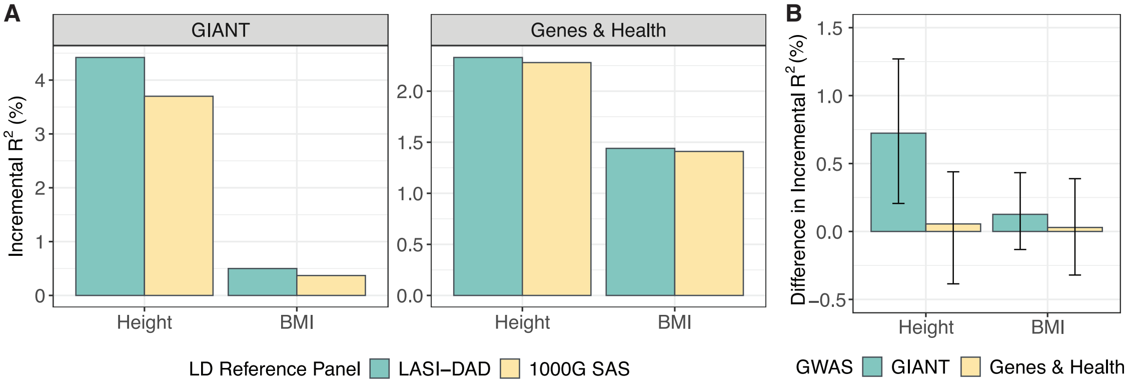

Figure 4. Polygenic risk score (PRS) analyses demonstrating the utility of LASI-DAD as an linkage disequilibrium (LD) reference panel

(A) The predictive performance of PRS, as measured by incremental R^2, was compared for PRS constructed with the LASI-DAD and 1000G South Asian (SAS) LD reference panels. Summary statistics for height and BMI were obtained from four studies of SAS ancestry, including a GWAS of height, an ExWAS of BMI from the GIANT consortium, and GWASs of both height and BMI from the Genes & Health study. PRS weights were estimated by PRS-CS.

(B) The improvement in the predictive performance of PRS constructed with the LASI-DAD reference panel relative to the 1000G SAS reference panel. Error bars indicate 95% confidence intervals estimated from bootstrapping with 1,000 resamples.

constructed with the 1000G SAS panel explained 2.28% and 1.41% of the variance in the corresponding traits. The improvement in R^2 was 0.05% (95% CI: −0.39% to 0.44%) for height and 0.03% (95% CI: −0.32% to 0.39%) for BMI. Although all estimates of the change in R^2 are in the positive direction, statistically significant differences were observed only for the PRS constructed using height summary statistics from the GIANT consortium. This is likely due to the inadequate sample sizes of the other three GWASs (ranging from 29,398 to 36,317) and the limited predictive power of their PRSs. Further evaluations will be warranted as additional SAS GWASs become available.

Providing LASI-DAD WGS data as an imputation reference panel

We evaluated the utility of LASI-DAD WGS data as an imputation reference panel and compared it with the GAsP and TOPMed imputation panels. Specifically, we found that the LASI-DAD panel enabled the imputation of many Indian-specific variants that are not present in the other panels, especially those with a low MAF (Figure 5A). For rare variants with an MAF ≤1%, the LASI-DAD panel was able to impute a total of 61,843,011 variants, representing 182% (21,948,408) and 940% (5,943,775) increases relative to the TOPMed and GAsP panels, respectively. Similarly, for common variants with an MAF >1%, the LASI-DAD panel was able to impute 8,783,797 variants, representing 4% (363,996) and 20% (1,449,768) increases relative to the TOPMed and GAsP panels. To ensure a fair comparison, we evaluated the imputation accuracy using the common set of variants that could be imputed by all three panels (Figure 5A). We found that the imputation with the LASI-DAD panel achieved substantially higher accuracy than with the other two panels for variants across most MAF ranges, except for singletons in the range of (0, 0.02)% (Figure 5B). For non-singletons, the LASI-DAD panel improved the imputation accuracy, as measured by Pearson correlation between imputed and true genotypes, by 3%–101% (mean 38%) compared with the TOPMed panel and by 3%–73% (mean 27%) compared with the GAsP panel across different MAF ranges. The imputation performance is consistent with the sample size of SAS individuals in each panel, with 1,654 samples in the GAsP panel and 644 in the TOPMed panel—both considerably smaller than that of the LASI-DAD panel. As expected, none of the panels can accurately impute singletons.

By combining imputation results from different reference panels, meta-imputation further improved the imputation accuracy, especially for variants with a low MAF (Figure 5B). Notably, meta-imputation using all three panels achieved the highest imputation accuracy across all MAF ranges. For variants with a relatively small MAF (≤0.1%), meta-imputation increased the accuracy by 14%–107% (mean 64%) compared with the best-performing reference panel. However, for variants with a relatively large MAF (>0.1%), incorporating additional reference panels beyond LASI-DAD offered only limited benefits, with imputation accuracy improving by 0.2%–2% (mean 0.8%) compared with the LASI-DAD panel. These results highlight the advantages of meta-imputation, in complementing the LASI-DAD panel, for imputing relatively rare variants in the Indian population.

Because it is standard practice to only carry forward variants with at least a moderate imputation quality score for downstream analyses, we examined the imputation accuracy for variants with an imputation quality score Rsq ≥0.3 to understand the practical impact of using the LASI-DAD panel for imputation compared to other panels. Additionally, given that Pearson correlation can be affected by allele frequency and is not a sensitive measure

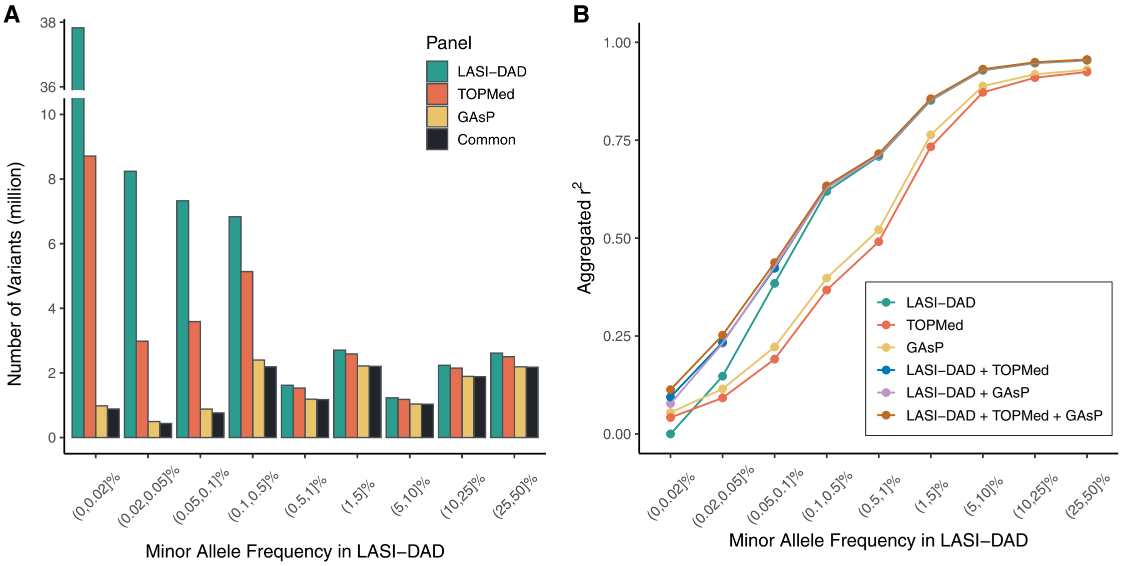

Figure 5. Evaluating LASI-DAD as a reference panel for genotype imputation
Compared reference panels include LASI-DAD, TOPMed, and GAsP.
(A) Bar plots showing the number of genetic variants in LASI-DAD that can be imputed by each reference panel and by all three panels.
(B) Imputation accuracy was evaluated for each reference panel, including meta-imputation to integrate the imputation results from either LASI-DAD and TOPMed, LASI-DAD and GAsP, or all three panels, across different MAF ranges. The MAF was calculated within the full 2,680 LASI-DAD samples. To ensure a fair comparison, the imputation accuracy was evaluated using the common set of variants that can be imputed by all three panels. Aggregated r^2 was used as the evaluation metric, calculated by grouping variants within a specified MAF range, stacking their imputed dosages and genotype calls from the sequencing data into two separate vectors, and then computing the squared correlation coefficient between the two vectors.

of imputation accuracy for rare variants, we also evaluated the imputation performance by calculating the NRD rate. The relative performance patterns of the different imputation reference panels remained largely similar to that observed when all variants were considered. Specifically, the LASI-DAD panel imputed a total of 14,985,203 variants of moderate quality suitable for downstream analyses, which is substantially higher than that imputed by the TOPMed (12,249,623 variants) and GAsP (8,614,897 variants) panels (Figure S6A). Importantly, the LASI-DAD panel also achieved higher imputation accuracy for these variants, as measured by both the Pearson correlation and NRD rate, than the TOPMed or GAsP panels (Figures S6B and S6C). Furthermore, meta-imputation provided additional improvements, particularly for rare variants with an MAF <0.1%.

Finally, we found that the imputation accuracy varied for samples from different states or territories of India, with the LASI-DAD panel exhibiting the smallest variation (Figure S7). This variation in imputation accuracy can be explained to some extent by the sample composition of the reference panels. For example, the TOPMed panel, which consists of nearly 50% EUR samples, exhibits a strong correlation between the imputation accuracy and the average %ANI for each state ($R^2 = 0.77$; Figure S8). This is consistent with the fact that samples with higher %ANI have closer relatedness to West Eurasians. In contrast, the correlation is much weaker with the LASI-DAD ($R^2 = 0.42$) or GAsP ($R^2 = 0.15$) panel. These results highlight the importance of having a large and nationally representative dataset for the Indian population to achieve the best imputation performance due to its complex population structure.

Discussion

As the largest and most nationally representative WGS study of the Indian population, LASI-DAD provides a unique opportunity to serve as a valuable resource for both LD and genotype imputation in this population. In the present study, we characterized the LD patterns in LASI-DAD by identifying LD blocks and evaluating regional differences in LD between the Indian population from LASI-DAD and four super-populations from the 1000G. Besides super-populations, we explored the population structure of India by evaluating LD patterns across Indian subpopulations. We evaluated the utility of LASI-DAD WGS data as an LD reference panel to facilitate both LD lookup and various statistical analyses that rely on precise LD estimates. Finally, we evaluated the utility of LASI-DAD as an imputation reference panel. As an LD reference panel, LASI-DAD provides a more comprehensive representation of genetic variation/structure in India and facilitates downstream LD-dependent analyses. As an imputation panel, it improves imputation accuracy compared with existing panels and demonstrates more robust performance across different Indian states or territories.

The comparison of LD patterns between LASI-DAD and four super-populations from 1000G confirms that the AFR

population experienced more recombination historically and has lower levels of long-range LD, as demonstrated by having the largest number of LD blocks and smallest overall block sizes.[52,54–57] In comparison, the EUR, EAS and SAS populations, all of which migrated from Africa, have similar levels of LD as indicated by a comparable number of blocks with similar block sizes.[58,59] As expected, the LD structure of LASI-DAD more closely resembles that of the 1000G SAS population than that of other worldwide populations, particularly with respect to the broad-scale LD blocks identified by LDetect. However, when examined using the fine-scale LD blocks identified by BigLD, we observed a smaller number of blocks with a larger average block size in LASI-DAD, suggesting stronger LD at the finer scale in LASI-DAD compared with the 1000G SAS. Our analysis also reveals remarkable heterogeneity in terms of pairwise LD estimates, as well as regions with considerable LD differences between LASI-DAD and 1000G SAS. This heterogeneity is further highlighted by global genetic PCs, showing that LASI-DAD significantly broadens the genetic boundaries typically seen in the 1000G SAS panel (Figure S9). Moreover, due to a significant increase in sample size, LASI-DAD interrogates significantly more (170%) variants, likely specific to the Indian/SAS population, that are not observed in the 1000G SAS panel. Given the improved representation and coverage, it is not surprising that the LASI-DAD panel, in comparison with the 1000G SAS panel, improves polygenic score construction and prediction in SAS. We expect similar improvement in other LD-based analyses that target Indian and SAS populations. Unfortunately, the sample sizes of SAS GWASs are limited; thus, the overall prediction accuracy of the corresponding PRS is much lower than that based on the EUR GWASs (Figure 2D vs. Figure 4). The next important step is to expand GWAS efforts in SAS populations, for which a good imputation reference panel is needed to generate high-quality genotype data. LASI-DAD can well serve that purpose as it improves imputation accuracy over existing popular reference panels for SASs, including TOPMed and GAsP. Importantly, it has more robust performance across all Indian states or territories, likely due to its unbiased representation of the Indian population. Our analysis further highlighted the potential advantage of leveraging multiple reference panels via meta-imputation to further improve imputation accuracy. However, it should be noted that various factors (e.g., size and composition of the panels, genotyping array density, phasing methods, allele frequency, frequency distribution across panels) can influence meta-imputation performance. It remains to be determined whether meta-imputation consistently outperforms single-panel imputation across all scenarios. All of these demonstrate that LASI-DAD is a valuable genetic reference panel for Indian and SAS populations.

The Indian population is diverse and shaped by multiple ancestral influences. Specifically, the majority of Indians are part of a north/south cline and are admixed with varying degrees of ANI (related to EUR, Central Asia, and Middle East populations) and ASI (related to endogenous ancestry). Meanwhile, a small proportion of Indians who are in the east have an additional ancestral influence from EAS. Our analysis by LASI-DAD sub-population confirms that this population structure leads to LD differences among sub-populations, with the largest difference observed between North Indians (high %ANI group) and East Indians (out-of-cline group), while South Indians (low %ANI group) are positioned in between. Since North Indians are most closely related to EUR, the predictive performance of EUR-based PRSs shows a similar gradient across these sub-populations, with the highest performance in North Indians, followed by South Indians and then East Indians. This is consistent with many studies, showing that the transferability of PRS decreases as the genetic distance between target population and discovery population increases.[60] The heterogeneity could also affect imputation accuracy across sub-populations when the reference panel is biased. This heterogeneity and potential consequence to health inequity underscore the importance of including diverse groups and having population-representative Indian samples, as exemplified by LASI-DAD.

While LASI-DAD has proven to be a valuable resource for serving as a reference panel, several opportunities are possible to further improve its utility. First, the current LASI-DAD panel is limited to bi-allelic variants. Future work should incorporate multi-allelic variants to enable more comprehensive and effective use of the resource. Second, while LASI-DAD represents the largest and most nationally representative WGS study, it remains insufficient to fully capture the extensive genetic diversity of the Indian population, which includes over 4,500 anthropologically defined groups. Substantial efforts to collect more samples are still needed to achieve a more comprehensive representation. Third, most existing genetic studies analyze SAS populations collectively as a single group. However, South Asia is a region that spans eight countries, making up $\sim$25% of the world's population and having a rich genetic diversity.[61] As a result, LD patterns observed in one SAS population may not accurately reflect those in another. This partly explains why the improvement in PRS performance when using LASI-DAD as the reference panel was smaller for the Genes & Health data than for the GIANT data, as the Genes & Health data consist of British Pakistani and British Bangladeshi, whereas a large proportion of individuals in the GIANT data are of Indian origin. To achieve the best results in statistical analyses with GWAS summary statistics, identifying the most appropriate reference panel with LD patterns that best match the GWAS population is critical. In line with this, an adaptive approach that combines multiple reference panels, akin to the meta-imputation approach, may provide an alternative for improving the LD reference panel. However, this would require the development of new statistical frameworks or analytical procedures.

In summary, we have presented LASI-DAD, the largest and most nationally representative WGS data from India, as a reference panel for LD and genotype imputation. This valuable resource fills a critical gap in the field of genetics and could greatly facilitate various genetic analyses in Indians and SAS. Meanwhile, the genetic diversity demonstrated in this study also underscores a critical need to expand data collection and enhance representativeness in this population.

Data and code availability

WGS data for the LASI-DAD are available from the National Institute on Aging Genetics of Alzheimer Disease Data Storage Site, accession number NG00067–ADSP Umbrella. Phenotype data are available at the Gateway to Global Aging website (https://g2aging.org/). The LASI-DAD LD reference panel is available at both GitHub (https://github.com/zhengli09/LASI_DAD-LD-Reference-Panel) and Zenodo (https://doi.org/10.5281/zenodo.17946912). The LASI-DAD imputation panel is available on the Michigan Imputation Server at https://imputationserver.sph.umich.edu.

Acknowledgments

We thank the LASI-DAD participants for being part of the research project. We thank Dr. Priya Moorjani at the University of California, Berkeley for her guidance on defining the LASI-DAD sub-populations. This study was supported by the National Institute on Aging (grant nos. R01 AG051125, U01 AG064948, and U54 AG052427). The funders had no role in study design, data collection and analysis, decision to publish, or preparation of the manuscript.

Author contributions

W.Z., J.A.S., and S.L.R.K. conceived the idea for the study. J.L. and S.L.R.K. provided funding support. A.B.D. and J.L. led the data collection in LASI-DAD. Y.Y.L., G.D.S., and L.-S.W. processed and cleaned the WGS data. L.F., S.S., and C.F. integrated LASI-DAD into the Michigan Imputation Server. Z.L. and W.Z. designed the experiments. Z.L. and W.Z. conducted the data analyses. Z.L., W.Z., and J.A.S. wrote the manuscript, with input from X.Z., Y.Y.L., and G.D.S. All authors read and approved the final manuscript.

Declaration of interests

The authors declare no competing interests.

Supplemental information

Supplemental information can be found online at https://doi.org/10.1016/j.xhgg.2026.100579.

Web resources

aggRSquare, https://github.com/yukt/aggRSquare
Eagle2.4, https://alkesgroup.broadinstitute.org/Eagle/#x1-130004
Genes & Health GWAS summary statistics, https://www.genesandhealth.org/research/gwas-data-downloads
GIANT consortium data files, https://giant-consortium.web.broadinstitute.org/index.php/GIANT_consortium_data_files
Gpart, https://mirror.accum.se/mirror/bioconductor.org/packages/3.14/bioc/vignettes/gpart/inst/doc/gpart.html
LD blocks identified from 1000G super-populations by LDetect, https://github.com/jmacdon/LDblocks_GRCh38
1000 Genomes 30× on GRCh38, https://www.internationalgenome.org/data-portal/data-collection/30x-grch38
PC-AiR and PC-Relate, https://bioconductor.posit.co/packages/3.19/bioc/vignettes/GENESIS/inst/doc/pcair.html
PRS-CS, https://github.com/getian107/PRScs
TOP-LD, http://topld.genetics.unc.edu/about.php

Received: July 16, 2025
Accepted: February 4, 2026

References

1. Mastana, S.S. (2014). Unity in diversity: an overview of the genomic anthropology of India. Ann. Hum. Biol. *41*, 287–299. https://doi.org/10.3109/03014460.2014.922615.
2. Reich, D., Thangaraj, K., Patterson, N., Price, A.L., and Singh, L. (2009). Reconstructing Indian population history. Nature *461*, 489–494. https://doi.org/10.1038/nature08365.
3. Moorjani, P., Thangaraj, K., Patterson, N., Lipson, M., Loh, P.R., Govindaraj, P., Berger, B., Reich, D., and Singh, L. (2013). Genetic evidence for recent population mixture in India. Am. J. Hum. Genet. *93*, 422–438. https://doi.org/10.1016/j.ajhg.2013.07.006.
4. Nakatsuka, N., Moorjani, P., Rai, N., Sarkar, B., Tandon, A., Patterson, N., Bhavani, G.S., Girisha, K.M., Mustak, M.S., Srinivasan, S., et al. (2017). The promise of discovering population-specific disease-associated genes in South Asia. Nat. Genet. *49*, 1403–1407. https://doi.org/10.1038/ng.3917.
5. Morales, J., Welter, D., Bowler, E.H., Cerezo, M., Harris, L.W., McMahon, A.C., Hall, P., Junkins, H.A., Milano, A., Hastings, E., et al. (2018). A standardized framework for representation of ancestry data in genomics studies, with application to the NHGRI-EBI GWAS Catalog. Genome Biol. *19*, 21. https://doi.org/10.1186/s13059-018-1396-2.
6. Sirugo, G., Williams, S.M., and Tishkoff, S.A. (2019). The Missing Diversity in Human Genetic Studies. Cell *177*, 26–31. https://doi.org/10.1016/j.cell.2019.02.048.
7. Wall, J.D., and Pritchard, J.K. (2003). Haplotype blocks and linkage disequilibrium in the human genome. Nat. Rev. Genet. *4*, 587–597. https://doi.org/10.1038/nrg1123.
8. Uffelmann, E., Huang, Q.Q., Munung, N.S., de Vries, J., Okada, Y., Martin, A.R., Martin, H.C., Lappalainen, T., and Posthuma, D. (2021). Genome-wide association studies. Nat Rev Method Prime *1*, 59. https://doi.org/10.1038/s43586-021-00056-9.
9. Schaid, D.J., Chen, W., and Larson, N.B. (2018). From genome-wide associations to candidate causal variants by statistical fine-mapping. Nat. Rev. Genet. *19*, 491–504. https://doi.org/10.1038/s41576-018-0016-z.
10. Li, Z., Zhao, W., Shang, L., Mosley, T.H., Kardia, S.L.R., Smith, J.A., and Zhou, X. (2022). METRO: Multi-ancestry transcriptome-wide association studies for powerful gene-trait association detection. Am. J. Hum. Genet. *109*, 783–801. https://doi.org/10.1016/j.ajhg.2022.03.003.
11. Kachuri, L., Chatterjee, N., Hirbo, J., Schaid, D.J., Martin, I., Kullo, I.J., Kenny, E.E., Pasaniuc, B., Polygenic Risk Methods

in Diverse Populations PRIMED Consortium Methods Working Group, Witte, J.S., and Ge, T. (2024). Principles and methods for transferring polygenic risk scores across global populations. Nat. Rev. Genet. *25*, 8–25. https://doi.org/10.1038/s41576-023-00637-2.

12. Gao, B., and Zhou, X. (2024). MESuSiE enables scalable and powerful multi-ancestry fine-mapping of causal variants in genome-wide association studies. Nat. Genet. *56*, 170–179. https://doi.org/10.1038/s41588-023-01604-7.
13. Marchini, J., and Howie, B. (2010). Genotype imputation for genome-wide association studies. Nat. Rev. Genet. *11*, 499–511. https://doi.org/10.1038/nrg2796.
14. Taliun, D., Harris, D.N., Kessler, M.D., Carlson, J., Szpiech, Z.A., Torres, R., Taliun, S.A.G., Corvelo, A., Gogarten, S.M., Kang, H.M., et al. (2021). Sequencing of 53,831 diverse genomes from the NHLBI TOPMed Program. Nature *590*, 290–299. https://doi.org/10.1038/s41586-021-03205-y.
15. Byrska-Bishop, M., Evani, U.S., Zhao, X., Basile, A.O., Abel, H.J., Regier, A.A., Corvelo, A., Clarke, W.E., Musunuri, R., Nagulapalli, K., et al. (2022). High-coverage whole-genome sequencing of the expanded 1000 Genomes Project cohort including 602 trios. Cell *185*, 3426–3440.e19. https://doi.org/10.1016/j.cell.2022.08.004.
16. Huang, L., Rosen, J.D., Sun, Q., Chen, J., Wheeler, M.M., Zhou, Y., Min, Y.I., Kooperberg, C., Conomos, M.P., Stilp, A.M., et al. (2022). TOP-LD: A tool to explore linkage disequilibrium with TOPMed whole-genome sequence data. Am. J. Hum. Genet. *109*, 1175–1181. https://doi.org/10.1016/j.ajhg.2022.04.006.
17. GenomeAsia100K Consortium (2019). The GenomeAsia 100K Project enables genetic discoveries across Asia. Nature *576*, 106–111. https://doi.org/10.1038/s41586-019-1793-z.
18. Lee, J., Khobragade, P.Y., Banerjee, J., Chien, S., Angrisani, M., Perianayagam, A., Bloom, D.E., and Dey, A.B. (2020). Design and Methodology of the Longitudinal Aging Study in India-Diagnostic Assessment of Dementia (LASI-DAD). J. Am. Geriatr. Soc. *68*, S5–S10. https://doi.org/10.1111/jgs.16737.
19. Zhao, W., Smith, J.A., Wang, Y.Z., Chintalapati, M., Ammous, F., Yu, M., Moorjani, P., Ganna, A., Gross, A., Dey, S., et al. (2023). Polygenic Risk Scores for Alzheimer's Disease and General Cognitive Function Are Associated With Measures of Cognition in Older South Asians. J. Gerontol. A Biol. Sci. Med. Sci. *78*, 743–752. https://doi.org/10.1093/gerona/glad057.
20. Morgulis, A., Gertz, E.M., Schäffer, A.A., and Agarwala, R. (2006). A fast and symmetric DUST implementation to mask low-complexity DNA sequences. J. Comput. Biol. *13*, 1028–1040. https://doi.org/10.1089/cmb.2006.13.1028.
21. Loh, P.R., Danecek, P., Palamara, P.F., Fuchsberger, C., A Reshef, Y., K Finucane, H., Schoenherr, S., Forer, L., McCarthy, S., Abecasis, G.R., et al. (2016). Reference-based phasing using the Haplotype Reference Consortium panel. Nat. Genet. *48*, 1443–1448. https://doi.org/10.1038/ng.3679.
22. Loh, P.R., Palamara, P.F., and Price, A.L. (2016). Fast and accurate long-range phasing in a UK Biobank cohort. Nat. Genet. *48*, 811–816. https://doi.org/10.1038/ng.3571.
23. Conomos, M.P., Miller, M.B., and Thornton, T.A. (2015). Robust inference of population structure for ancestry prediction and correction of stratification in the presence of relatedness. Genet. Epidemiol. *39*, 276–293. https://doi.org/10.1002/gepi.21896.
24. Wang, Y.Z., Zhao, W., Moorjani, P., Gross, A.L., Zhou, X., Dey, A.B., Lee, J., Smith, J.A., and Kardia, S.L.R. (2024). Effect of apolipoprotein E epsilon4 and its modification by sociodemographic characteristics on cognitive measures in South Asians from LASI-DAD. Alzheimer's Dement. *20*, 4854–4867. https://doi.org/10.1002/alz.14052.
25. Conomos, M.P., Reiner, A.P., Weir, B.S., and Thornton, T.A. (2016). Model-free Estimation of Recent Genetic Relatedness. Am. J. Hum. Genet. *98*, 127–148. https://doi.org/10.1016/j.ajhg.2015.11.022.
26. Ge, T., Chen, C.Y., Ni, Y., Feng, Y.C.A., and Smoller, J.W. (2019). Polygenic prediction via Bayesian regression and continuous shrinkage priors. Nat. Commun. *10*, 1776. https://doi.org/10.1038/s41467-019-09718-5.
27. Zhang, J., Zhan, J., Jin, J., Ma, C., Zhao, R., O'Connell, J., Jiang, Y., 23andMe Research Team, Koelsch, B.L., Zhang, H., and Chatterjee, N. (2024). An ensemble penalized regression method for multi-ancestry polygenic risk prediction. Nat. Commun. *15*, 3238. https://doi.org/10.1038/s41467-024-47357-7.
28. Zhang, H., Zhan, J., Jin, J., Zhang, J., Lu, W., Zhao, R., Ahearn, T.U., Yu, Z., O'Connell, J., Jiang, Y., et al. (2023). A new method for multiancestry polygenic prediction improves performance across diverse populations. Nat. Genet. *55*, 1757–1768. https://doi.org/10.1038/s41588-023-01501-z.
29. Werme, J., van der Sluis, S., Posthuma, D., and de Leeuw, C.A. (2022). An integrated framework for local genetic correlation analysis. Nat. Genet. *54*, 274–282. https://doi.org/10.1038/s41588-022-01017-y.
30. Zhang, Y., Lu, Q., Ye, Y., Huang, K., Liu, W., Wu, Y., Zhong, X., Li, B., Yu, Z., Travers, B.G., et al. (2021). SUPERGNOVA: local genetic correlation analysis reveals heterogeneous etiologic sharing of complex traits. Genome Biol. *22*, 262. https://doi.org/10.1186/s13059-021-02478-w.
31. Li, Y., Pawitan, Y., and Shen, X. (2025). An enhanced framework for local genetic correlation analysis. Nat. Genet. *57*, 1053–1058. https://doi.org/10.1038/s41588-025-02123-3.
32. Berisa, T., and Pickrell, J.K. (2016). Approximately independent linkage disequilibrium blocks in human populations. Bioinformatics *32*, 283–285. https://doi.org/10.1093/bioinformatics/btv546.
33. Wen, X., and Stephens, M. (2010). Using Linear Predictors to Impute Allele Frequencies from Summary or Pooled Genotype Data. Ann. Appl. Stat. *4*, 1158–1182. https://doi.org/10.1214/10-aoas338.
34. MacDonald, J.W., Harrison, T.A., Bammler, T.K., Mancuso, N., and Lindström, S. (2023). Ancestry-specific maps of GRCh38 linkage disequilibrium blocks for human genome research. Preprint at bioRxiv. https://doi.org/10.1101/2022.03.04.483057.
35. Spence, J.P., and Song, Y.S. (2019). Inference and analysis of population-specific fine-scale recombination maps across 26 diverse human populations. Sci. Adv. *5*, eaaw9206. https://doi.org/10.1126/sciadv.aaw9206.
36. Kim, S.A., Cho, C.S., Kim, S.R., Bull, S.B., and Yoo, Y.J. (2018). A new haplotype block detection method for dense genome sequencing data based on interval graph modeling of clusters of highly correlated SNPs. Bioinformatics *34*, 388–397. https://doi.org/10.1093/bioinformatics/btx609.
37. Teo, Y.Y., Fry, A.E., Bhattacharya, K., Small, K.S., Kwiatkowski, D.P., and Clark, T.G. (2009). Genome-wide comparisons of variation in linkage disequilibrium. Genome Res. *19*, 1849–1860. https://doi.org/10.1101/gr.092189.109.

38. Kerdoncuff, E., Skov, L., Patterson, N., Banerjee, J., Khobragade, P., Chakrabarti, S.S., Chakrawarty, A., Chatterjee, P., Dhar, M., Gupta, M., et al. (2025). 50,000 years of evolutionary history of India: Impact on health and disease variation. Cell *188*, 3389–3404.e6. https://doi.org/10.1016/j.cell.2025.04.027.
39. Maier, R., Flegontov, P., Flegontova, O., Işıldak, U., Changmai, P., and Reich, D. (2023). On the limits of fitting complex models of population history to f-statistics. eLife *12*, e85492. https://doi.org/10.7554/eLife.85492.
40. Martin, A.R., Kanai, M., Kamatani, Y., Okada, Y., Neale, B.M., and Daly, M.J. (2019). Clinical use of current polygenic risk scores may exacerbate health disparities. Nat. Genet. *51*, 584–591. https://doi.org/10.1038/s41588-019-0379-x.
41. Yengo, L., Vedantam, S., Marouli, E., Sidorenko, J., Bartell, E., Sakaue, S., Graff, M., Eliasen, A.U., Jiang, Y., Raghavan, S., et al. (2022). A saturated map of common genetic variants associated with human height. Nature *610*, 704–712. https://doi.org/10.1038/s41586-022-05275-y.
42. Yengo, L., Sidorenko, J., Kemper, K.E., Zheng, Z., Wood, A.R., Weedon, M.N., Frayling, T.M., Hirschhorn, J., Yang, J., Visscher, P.M.; and GIANT Consortium (2018). Meta-analysis of genome-wide association studies for height and body mass index in approximately 700000 individuals of European ancestry. Hum. Mol. Genet. *27*, 3641–3649. https://doi.org/10.1093/hmg/ddy271.
43. Zou, Y., Carbonetto, P., Wang, G., and Stephens, M. (2022). Fine-mapping from summary data with the "Sum of Single Effects" model. PLoS Genet. *18*, e1010299. https://doi.org/10.1371/journal.pgen.1010299.
44. Zhou, X. (2017). A Unified Framework for Variance Component Estimation with Summary Statistics in Genome-Wide Association Studies. Ann. Appl. Stat. *11*, 2027–2051. https://doi.org/10.1214/17-AOAS1052.
45. Mancuso, N., Freund, M.K., Johnson, R., Shi, H., Kichaev, G., Gusev, A., and Pasaniuc, B. (2019). Probabilistic fine-mapping of transcriptome-wide association studies. Nat. Genet. *51*, 675–682. https://doi.org/10.1038/s41588-019-0367-1.
46. Li, Z., Gao, B., and Zhou, X. (2025). An alternative framework for transcriptome-wide association studies to detect and decipher gene-trait associations. Preprint at bioRxiv. https://doi.org/10.1101/2025.03.14.643391.
47. Turcot, V., Lu, Y., Highland, H.M., Schurmann, C., Justice, A.E., Fine, R.S., Bradfield, J.P., Esko, T., Giri, A., Graff, M., et al. (2018). Protein-altering variants associated with body mass index implicate pathways that control energy intake and expenditure in obesity. Nat. Genet. *50*, 26–41. https://doi.org/10.1038/s41588-017-0011-x.
48. Finer, S., Martin, H.C., Khan, A., Hunt, K.A., MacLaughlin, B., Ahmed, Z., Ashcroft, R., Durham, C., MacArthur, D.G., McCarthy, M.I., et al. (2020). Cohort Profile: East London Genes & Health (ELGH), a community-based population genomics and health study in British Bangladeshi and British Pakistani people. Int. J. Epidemiol. *49*, 20–21i. https://doi.org/10.1093/ije/dyz174.
49. Yu, K., Das, S., LeFaive, J., Kwong, A., Pleiness, J., Forer, L., Schönherr, S., Fuchsberger, C., Smith, A.V., and Abecasis, G.R. (2022). Meta-imputation: An efficient method to combine genotype data after imputation with multiple reference panels. Am. J. Hum. Genet. *109*, 1007–1015. https://doi.org/10.1016/j.ajhg.2022.04.002.
50. Hofmeister, R.J., Ribeiro, D.M., Rubinacci, S., and Delaneau, O. (2023). Accurate rare variant phasing of whole-genome and whole-exome sequencing data in the UK Biobank. Nat. Genet. *55*, 1243–1249. https://doi.org/10.1038/s41588-023-01415-w.
51. Das, S., Forer, L., Schönherr, S., Sidore, C., Locke, A.E., Kwong, A., Vrieze, S.I., Chew, E.Y., Levy, S., McGue, M., et al. (2016). Next-generation genotype imputation service and methods. Nat. Genet. *48*, 1284–1287. https://doi.org/10.1038/ng.3656.
52. Campbell, M.C., and Tishkoff, S.A. (2008). African genetic diversity: implications for human demographic history, modern human origins, and complex disease mapping. Annu. Rev. Genomics Hum. Genet. *9*, 403–433. https://doi.org/10.1146/annurev.genom.9.081307.164258.
53. Koenig, Z., Yohannes, M.T., Nkambule, L.L., Zhao, X., Goodrich, J.K., Kim, H.A., Wilson, M.W., Tiao, G., Hao, S.P., Sahakian, N., et al. (2024). A harmonized public resource of deeply sequenced diverse human genomes. Genome Res. *34*, 796–809. https://doi.org/10.1101/gr.278378.123.
54. Gabriel, S.B., Schaffner, S.F., Nguyen, H., Moore, J.M., Roy, J., Blumenstiel, B., Higgins, J., DeFelice, M., Lochner, A., Faggart, M., et al. (2002). The structure of haplotype blocks in the human genome. Science *296*, 2225–2229. https://doi.org/10.1126/science.1069424.
55. Lonjou, C., Zhang, W., Collins, A., Tapper, W.J., Elahi, E., Maniatis, N., and Morton, N.E. (2003). Linkage disequilibrium in human populations. Proc. Natl. Acad. Sci. USA *100*, 6069–6074. https://doi.org/10.1073/pnas.1031521100.
56. Reich, D.E., Cargill, M., Bolk, S., Ireland, J., Sabeti, P.C., Richter, D.J., Lavery, T., Kouyoumjian, R., Farhadian, S.F., Ward, R., and Lander, E.S. (2001). Linkage disequilibrium in the human genome. Nature *411*, 199–204. https://doi.org/10.1038/35075590.
57. Sawyer, S.L., Mukherjee, N., Pakstis, A.J., Feuk, L., Kidd, J.R., Brookes, A.J., and Kidd, K.K. (2005). Linkage disequilibrium patterns vary substantially among populations. Eur. J. Hum. Genet. *13*, 677–686. https://doi.org/10.1038/sj.ejhg.5201368.
58. De La Vega, F.M., Isaac, H., Collins, A., Scafe, C.R., Halldórsson, B.V., Su, X., Lippert, R.A., Wang, Y., Laig-Webster, M., Koehler, R.T., et al. (2005). The linkage disequilibrium maps of three human chromosomes across four populations reflect their demographic history and a common underlying recombination pattern. Genome Res. *15*, 454–462. https://doi.org/10.1101/gr.3241705.
59. Pemberton, T.J., Jakobsson, M., Conrad, D.F., Coop, G., Wall, J.D., Pritchard, J.K., Patel, P.I., and Rosenberg, N.A. (2008). Using population mixtures to optimize the utility of genomic databases: linkage disequilibrium and association study design in India. Ann. Hum. Genet. *72*, 535–546. https://doi.org/10.1111/j.1469-1809.2008.00457.x.
60. Ziyatdinov, A., Torres, J., Alegre-Díaz, J., Backman, J., Mbatchou, J., Turner, M., Gaynor, S.M., Joseph, T., Zou, Y., Liu, D., et al. (2023). Genotyping, sequencing and analysis of 140,000 adults from Mexico City. Nature *622*, 784–793. https://doi.org/10.1038/s41586-023-06595-3.
61. Dokuru, D.R., Horwitz, T.B., Freis, S.M., Stallings, M.C., and Ehringer, M.A. (2024). South Asia: The Missing Diverse in Diversity. Behav. Genet. *54*, 51–62. https://doi.org/10.1007/s10519-023-10161-y.

HGGA, Volume 7

Supplemental information

A reference panel for linkage disequilibrium and genotype imputation using whole-genome sequencing data from 2,680 participants across India

Zheng Li, Wei Zhao, Xiang Zhou, Yuk Yee Leung, Gerard D. Schellenberg, Li-San Wang, Sebastian Schönherr, Lukas Forer, Christian Fuchsberger, Sharmistha Dey, Jinkook Lee, Jennifer A. Smith, Aparajit B. Dey, and Sharon L.R. Kardia

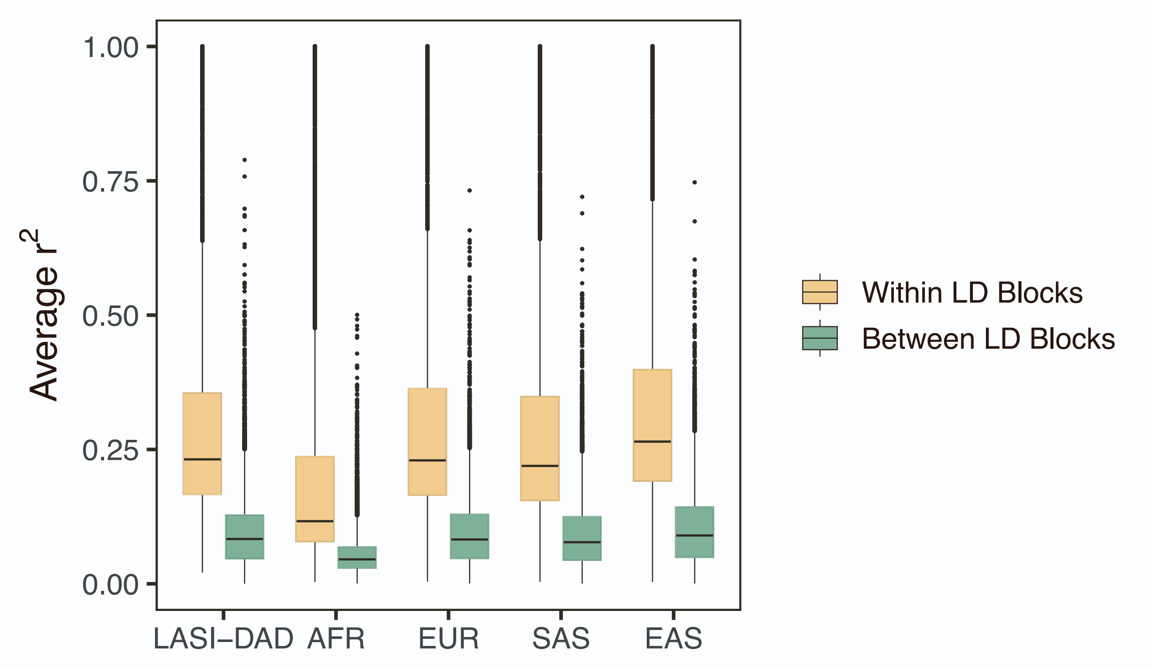

Figure S1. Distribution of average linkage disequilibrium (LD) on chromosome 1. The average LD was evaluated either between all pairs of SNPs within each LD block or between SNPs in each LD block and its adjacent two blocks. LD blocks were identified by BigLD at fine scales. LD was evaluated as the squared correlation coefficient (r^2) of genotypes between a SNP pair.

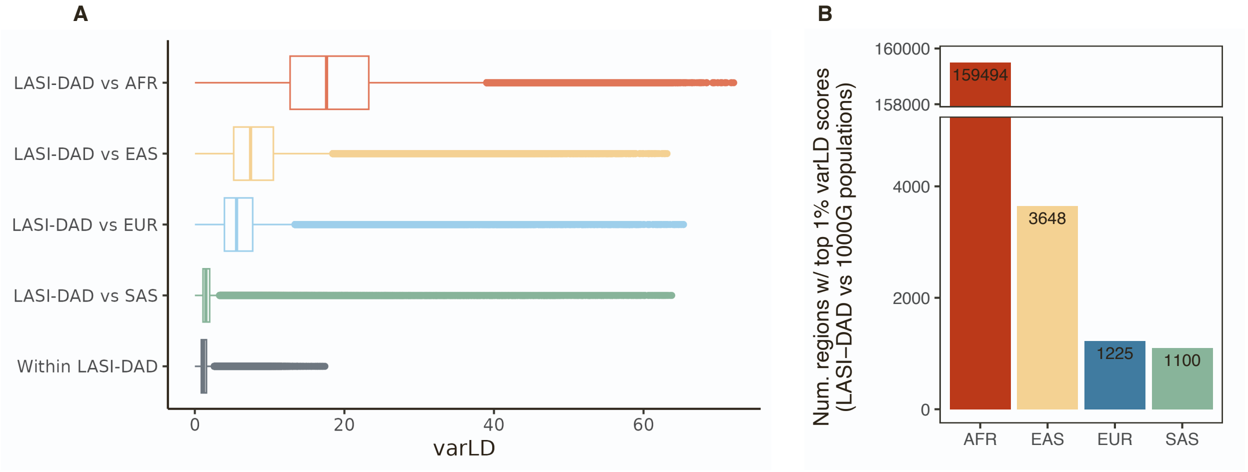

Figure S2. **Extensive differences in linkage disequilibrium (LD) patterns between LASI-DAD and other populations demonstrated by the variation in LD (varLD) scores**. (**A**) Boxplots showing the distribution of varLD scores evaluated between LASI-DAD and each of the four super-populations from 1000G, as well as between two random splits of LASI-DAD samples. The two splits of samples were obtained by randomly selecting 500 samples as the first split and assigning the remaining samples to the second split. (**B**) Number of genomic regions within the top one percentile of the varLD scores across all comparisons between LASI-DAD and each of the four 1000G super-populations.

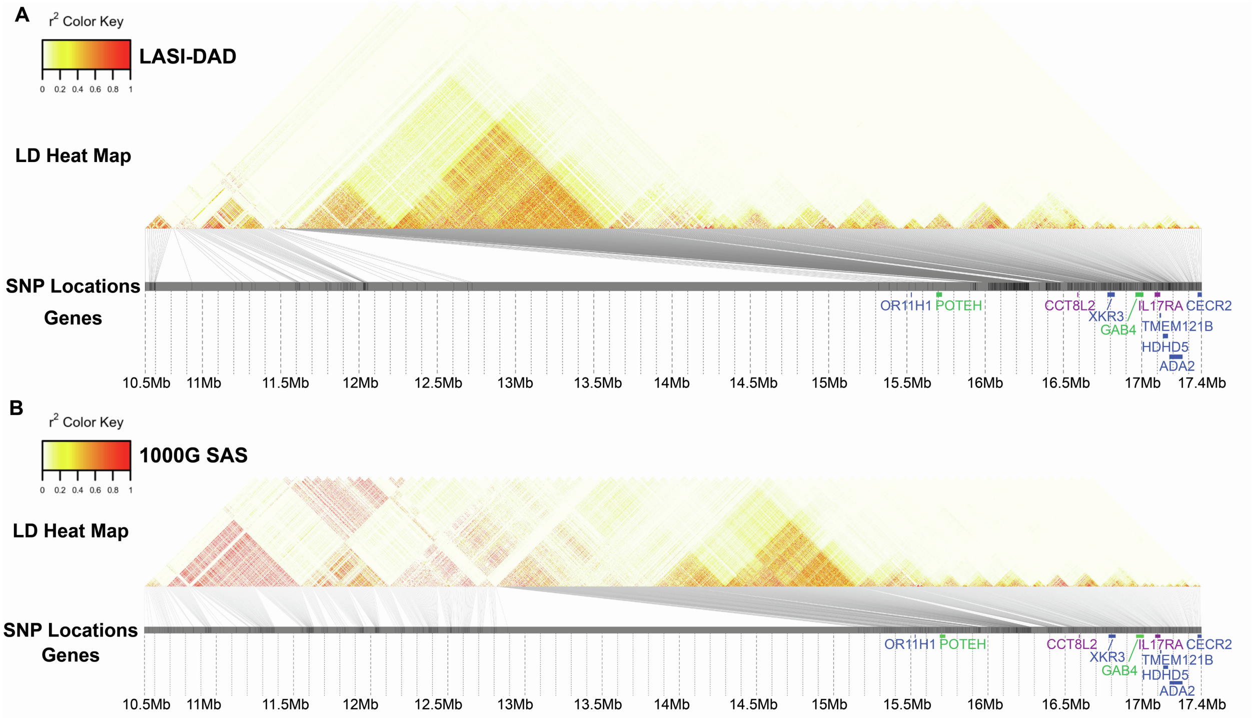

Figure S3. Linkage disequilibrium (LD) patterns of an example genomic region. Visualization of an example LD block identified by LDetect from LASI-DAD in the (**A**) LASI-DAD or (**B**) 1000G SAS population. The LD block spans the region from 10,526,445 to 17,381,714 on chromosome 22 and was selected because it contains the genomic region with the largest variation in LD (varLD) score between LASI-DAD and the 1000G SAS population on this chromosome (varLD = 21.7).

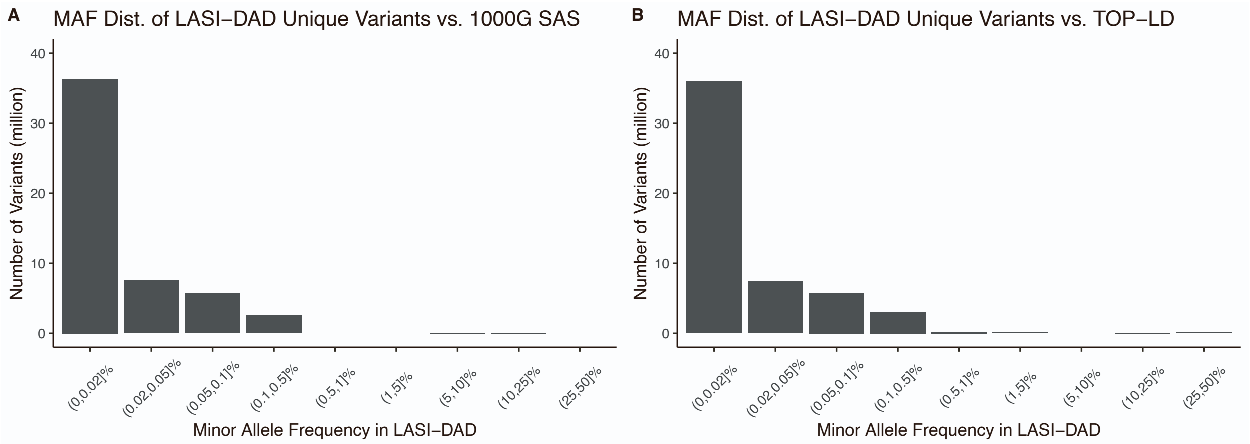

Figure S4. Barplots showing the number of unique variants in the LASI-DAD linkage disequilibrium (LD) lookup panel compared with the (**A**) 1000G SAS panel and (**B**) TOP-LD SAS panel across different minor allele frequency (MAF) ranges.

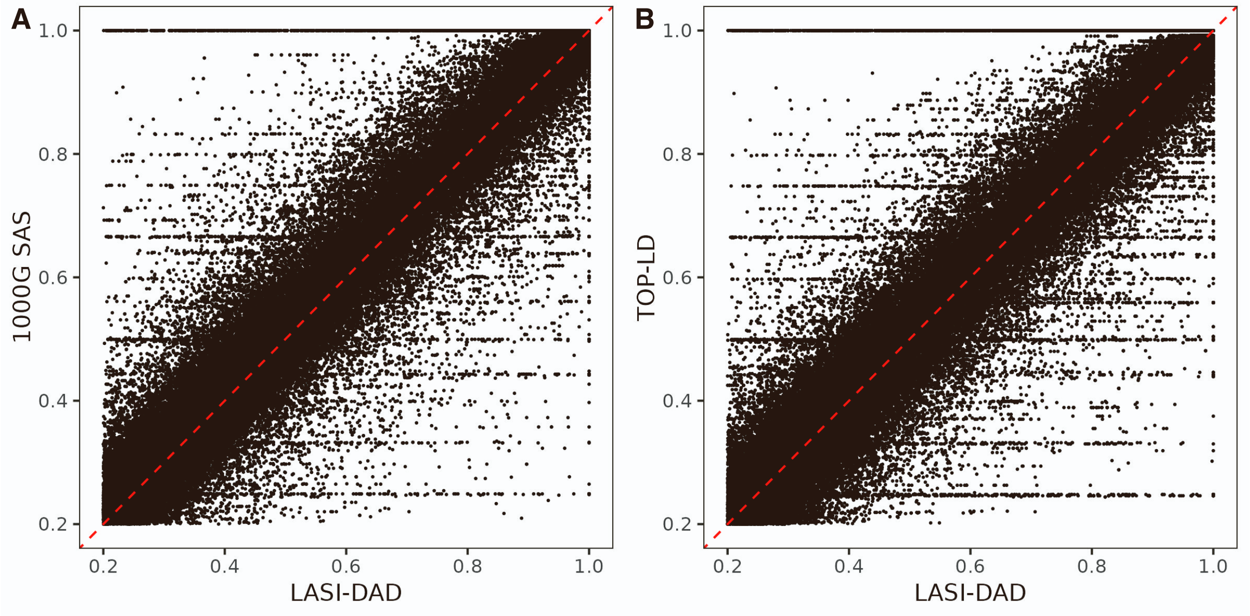

Figure S5. Comparing the linkage disequilibrium (LD) lookup panels constructed with different populations. Scatterplots comparing the r^2 estimates between (A) LASI-DAD and 1000G SAS LD panels and (B) LASI-DAD and TOP-LD SAS LD panels. For computational efficiency, 100,000 variant pairs shared between the two panels were randomly selected from chromosome 1 for the comparison.

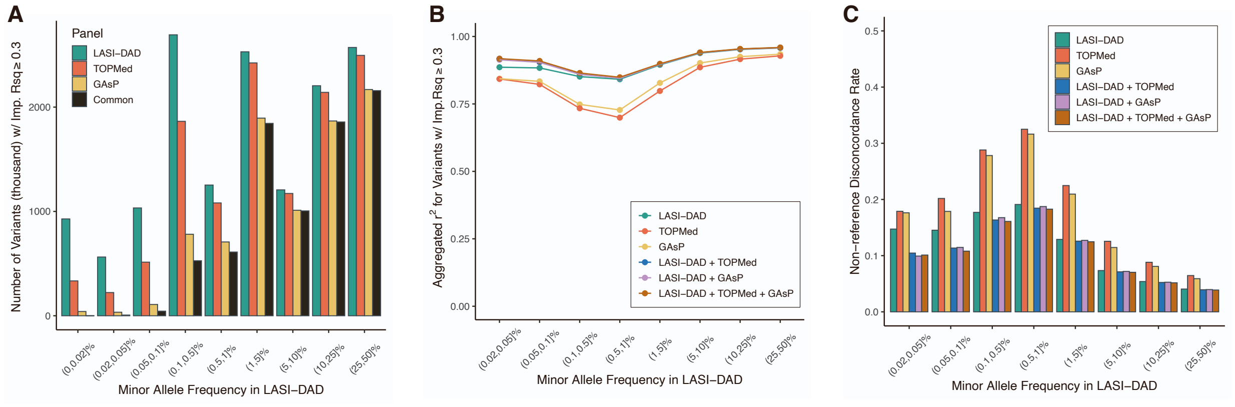

Figure S6. Evaluating LASI-DAD as a reference panel for genotype imputation for variants with an estimated imputation quality score Rsq ≥ 0.3. Compared reference panels include LASI-DAD, TOPMed, and GAsP. (**A**) Bar plots showing the number of genetic variants in LASI-DAD that can be imputed by each reference panel and by all three panels. (**B**) Imputation accuracy was evaluated for each reference panel, including meta-imputation to integrate the imputation results from either LASI-DAD and TOPMed, LASI-DAD and GAsP, or all three panels, across different minor allele frequency (MAF) ranges. The MAF was calculated within the full 2,680 LASI-DAD samples. To ensure a fair comparison, the imputation accuracy was evaluated using the common set of variants that can be imputed by all three panels. Aggregated r^2 was used as the evaluation metric, calculated by grouping variants within a specified MAF range, stacking their imputed dosages and genotype calls from the sequencing data into two separate vectors, and then computing the squared correlation coefficient between the two vectors. (**C**) Imputation accuracy was evaluated based on the nonreference discordance (NRD) rate.

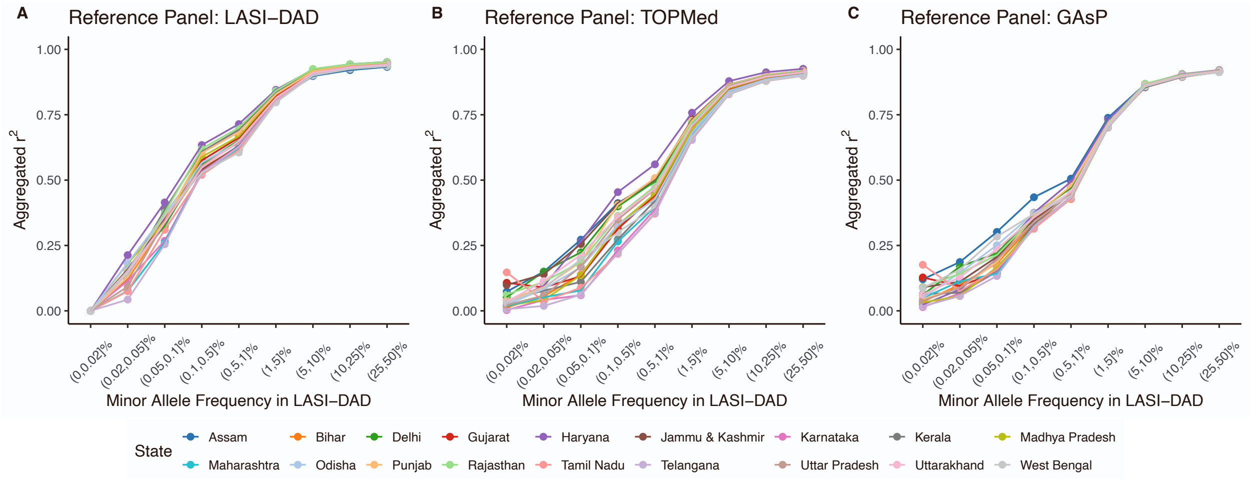

Figure S7. Imputation accuracy by Indian state/territory. Imputation accuracy was evaluated for samples from each Indian state or union territory across different minor allele frequency (MAF) ranges. Compared imputation reference panels include LASI-DAD, TOPMed, and GAsP. The MAF was calculated within the full 2,680 LASI-DAD samples. To ensure a fair comparison, the imputation accuracy was evaluated using the common set of variants that can be imputed by all three panels. Aggregated r^2 was used as the evaluation metric, calculated by grouping variants within a specified MAF range, stacking their imputed dosages and genotype calls from the sequencing data into two separate vectors, and then computing the squared correlation coefficient between the two vectors.

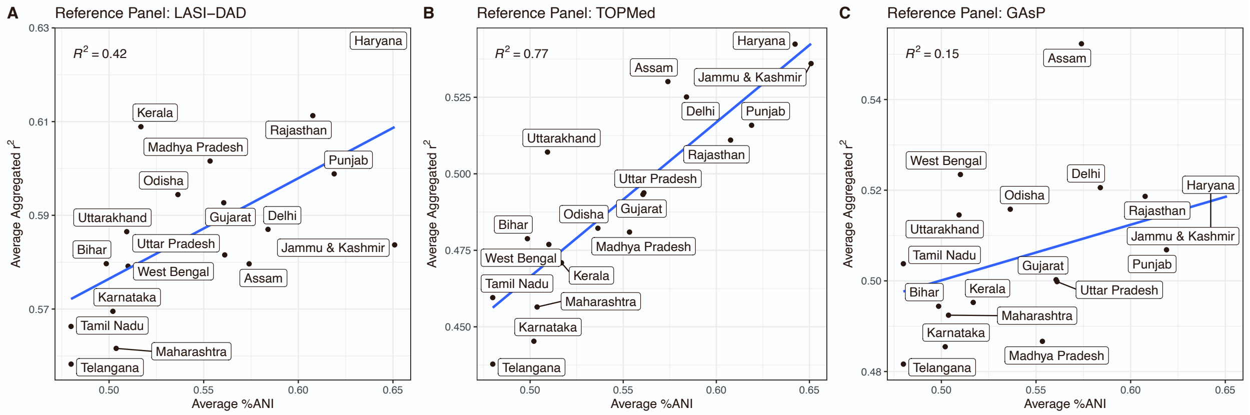

Figure S8. Average proportion of Ancestral North Indian (%ANI) in each state/territory of India versus average aggregated imputation r^2 with different panels. The aggregated r^2 was computed with imputed dosages from panel (**A**) LASI-DAD, (**B**) TOPMed, or (**C**) GAsP.

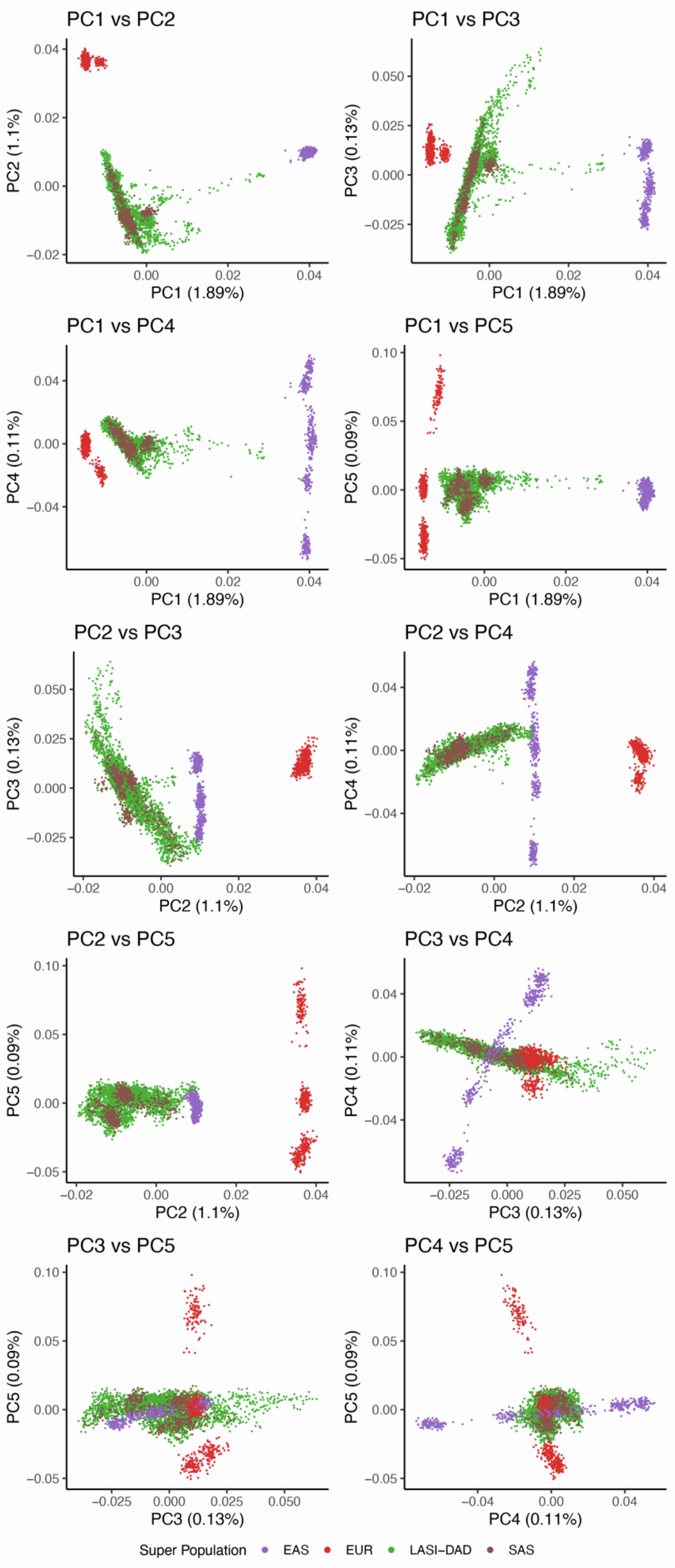

PC1 vs PC2
PC1 vs PC3
PC1 vs PC4
PC1 vs PC5
PC2 vs PC3
PC2 vs PC4
PC2 vs PC5
PC3 vs PC4
PC3 vs PC5
PC4 vs PC5
PC1 (1.89%)
PC2 (1.1%)
PC3 (0.13%)
PC4 (0.11%)
PC5 (0.09%)
Super Population
EAS
EUR
LASI-DAD
SAS

Figure S9. Principal component analysis (PCA) plots. PCA was conducted using Indian samples from LASI-DAD along with European, South Asian, and East Asian samples from 1000G. The proportion of variance explained by each PC is indicated in the parentheses.

Geographic region of India	State/Territory	Sample size
North	Jammu & Kashmir	90
	Punjab	112
	Haryana	171
	Delhi	120
	Rajasthan	151
Northeast	Assam	72
East	Bihar	137
	West Bengal	187
	Odisha	216
Central	Uttarakhand	68
	Uttar Pradesh	154
	Madhya Pradesh	77
West	Gujarat	228
	Maharashtra	185
South	Telangana	171
	Karnataka	122
	Tamil Nadu	159
	Kerala	260

Table S1. Sample sizes of Indian states and union territories by geographic region in LASI-DAD.
